# Macrophages and nociceptor neurons form a sentinel unit around fenestrated capillaries to defend the synovium from circulating immune challenge

**DOI:** 10.1038/s41590-024-02011-8

**Published:** 2024-11-25

**Authors:** Tetsuo Hasegawa, Colin Y. C. Lee, Andrew J. Hotchen, Aaron Fleming, Rahul Singh, Kunimichi Suzuki, Michisuke Yuzaki, Masahiko Watanabe, Mark A. Birch, Andrew W. McCaskie, Nikolett Lénárt, Krisztina Tóth, Ádám Dénes, Zhaoyuan Liu, Florent Ginhoux, Nathan Richoz, Menna R. Clatworthy

**Affiliations:** 1 Molecular Immunity Unit, University of Cambridge, Department of Medicine, Medical Research Council Laboratory of Molecular Biology, Cambridge, UK; 2 Cambridge Institute for Therapeutic Immunology and Infectious Diseases, University of Cambridge, Cambridge, UK; 3 Cellular Genetics, Wellcome Sanger Institute, Hinxton, UK; 4 Division of Trauma and Orthopaedic Surgery, Department of Surgery, University of Cambridge, Cambridge, UK; 5 Department of Physiology, Keio University School of Medicine, Tokyo, Japan; 6 Department of Anatomy, Faculty of Medicine, Hokkaido University, Sapporo, Japan; 7 Momentum Laboratory of Neuroimmunology, Institute of Experimental Medicine, Budapest, Hungary; 8 Shanghai Institute of Immunology, Shanghai Jiao Tong University School of Medicine, Shanghai, China; 9 INSERM U1015, Gustave Roussy, Villejuif, France

**Keywords:** Innate immunity, Monocytes and macrophages, Neuroimmunology, Systemic lupus erythematosus

## Abstract

A wide variety of systemic pathologies, including infectious and autoimmune diseases, are accompanied by joint pain or inflammation, often mediated by circulating immune complexes (ICs). How such stimuli access joints and trigger inflammation is unclear. Whole-mount synovial imaging revealed PV1^+^ fenestrated capillaries at the periphery of the synovium in the lining–sublining interface. Circulating ICs extravasated from these PV1^+^ capillaries, and nociceptor neurons and three distinct macrophage subsets formed a sentinel unit around them. Macrophages showed subset-specific responses to systemic IC challenge; LYVE1^+^CX_3_CR1^+^ macrophages orchestrated neutrophil recruitment and activated calcitonin gene-related peptide^+^ (CGRP^+^) nociceptor neurons via interleukin-1β. In contrast, major histocompatibility complex class II^+^CD11c^+^ (MHCII^+^CD11c^+^) and MHCII^+^CD11c^–^ interstitial macrophages formed tight clusters around PV1^+^ capillaries in response to systemic immune stimuli, a feature enhanced by nociceptor-derived CGRP. Altogether, we identify the anatomical location of synovial PV1^+^ capillaries and subset-specific macrophage–nociceptor cross-talk that forms a blood–joint barrier protecting the synovium from circulating immune challenges.

## Abstract (web summary)

Why joints are highly responsive to systemic inflammation is unknown. Hasegawa et al. sought to address this question, developing a whole-mount imaging system of the entire synovium to profile the vascular, neuronal and immune components.

## Main

Synovial joints are the target of antigen-specific autoimmune responses in diseases such as rheumatoid arthritis (RA)^1^, but joint pain (arthralgia), or even overt inflammation, is a relatively common manifestation of viral and bacterial infections in distant unrelated organs (for example, bacterial enteritis or streptococcal pharyngitis). This phenomenon is thought, at least in part, to be mediated by circulating microbial antigen–IgG immune complexes (ICs)^2–4^. Similarly, in systemic lupus erythematosus, an autoimmune disease characterized by IgG IC deposition, arthritis is a common symptom^5^. Thus, synovial joints act as a ‘barometer’ of systemic inflammation, manifesting as arthralgia or arthritis.

The synovial membrane lines the joint cavity, is the source of synovial fluid and contains several fibroblast and macrophage subsets^6,7^. It is highly vascular, supplies oxygen and nutrients to adjacent avascular articular cartilage and contains pain fibers^8–12^. The synovium is also involved in joint pathology, with proliferative pannus arising at the synovia–bone interface, eroding bone in RA^13–15^.

There is increasing interest in the interplay between neuronal signals and tissue immune cells^16–18^, but whether or how the triggering of arthralgia-generating pain neurons in response to circulating stimuli is influenced by synovial immune cells or how immune cells in turn might be affected by pain is currently unknown. Here, we established a whole-mount imaging system for the synovium to gain a comprehensive understanding of the spatial organization of the constituent immune, vascular and neuronal components. We investigated the responses of these components to circulating stimuli and addressed the question of why joints are responsive to many systemic pathologies.

## Results

### PV1^+^ fenestrated capillaries at the periphery of the synovium allow access to circulating stimuli

**Fig. 1:**
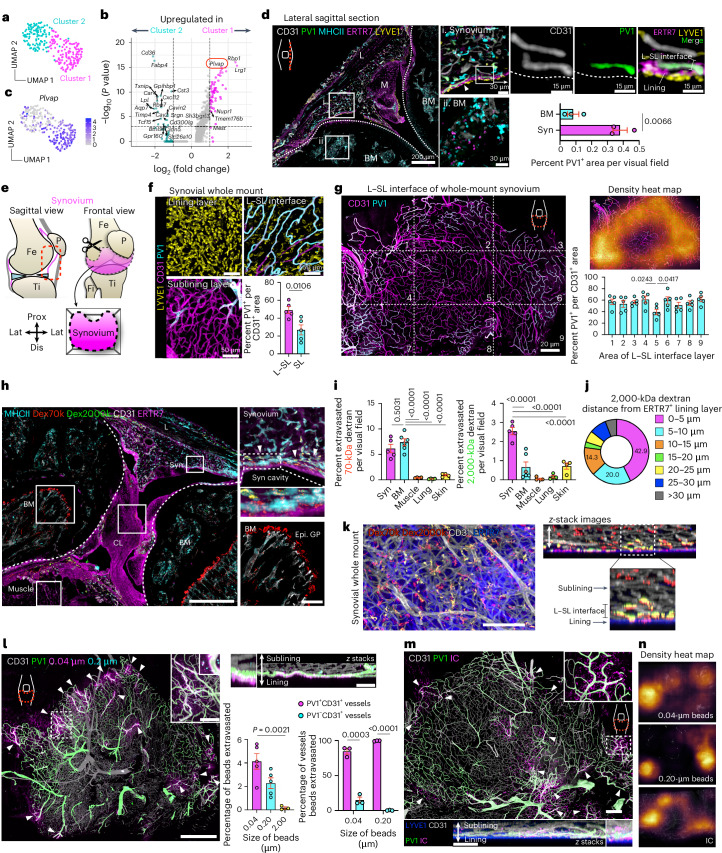
PV1^+^ fenestrated capillaries in the L–SL interface at the peripheral area of the synovium allow circulating stimuli to access the synovium. **a**, Uniform manifold approximation and projection (UMAP) visualization of synovial capillary endothelial cells extracted from CD31^+^ endothelial cells (Extended Data Fig. 1a,b). scRNA-seq data are from GSE145286. **b**, Volcano plots showing DEGs between two clusters of synovial capillary endothelial cells. **c**, UMAP visualization of synovial capillary endothelial cells expressing *Plvap*. Color bar shows the expression level. **d**, Representative confocal images of sections of knee joints; BM, bone marrow; M, meniscus; L, patella ligament; Syn, synovium; *n* = 3 mice for each group. **e**, Schematic diagram showing the protocol to dissect whole synovium from knee joints. The red dashed outline indicates the area of synovium dissected. Fe, femur; Ti, tibia; P, patella; Fi, fibula; Prox, proximal; Lat, lateral; Dis, distal. **f**, Three-dimensional reconstruction of representative confocal images of the indicated layer of whole-mount synovium. Quantification of the PV1^+^ area among CD31^+^ area at the indicated layers is shown on the bottom right; *n* = 5 mice for each group. **g**, Three-dimensional reconstruction of representative confocal images and density map of the L–SL interface of whole-mount synovium. Quantification of the PV1^+^ area among CD31^+^ area at the indicated compartments is shown on the bottom right; *n* = 5 mice. **h**, Representative confocal images of sections of knee joints from WT mice injected i.v. with 70- and 2,000-kDa dextran (300 μg of 70-kDa dextran (Dex70k) and 150 μg of 2,000-kDa dextran (Dex2000k)) 1 h before analysis. Arrowheads indicate the area where 70- and 2,000-kDa dextran merged; CL, cruciate ligament; Epi. GP, epiphyseal growth plate; scale bars, 500 and 100 μm (inset). **i**, Quantification of the extravasated area in each tissue; *n* = 4 to 6 mice for each group. **j**, Pie graph showing the percentage of distance between 2,000-kDa dextran and the ERTR7^+^ lining layer of the synovium in the section images; *n* = 4 mice. **k**, Three-dimensional reconstruction of representative confocal images of whole-mount synovium from WT mice injected i.v. with 70- and 2,000-kDa dextran 1 h before analysis; scale bar, 100 μm. **l**, Three-dimensional reconstruction of representative confocal images of whole-mount synovium from WT mice injected i.v. with fluorescently labeled microbeads of different sizes (25 μl of each FluoSphere carboxylate-modified microsphere dissolved in PBS) 1 h before analysis. Arrowheads indicate the sites where microbeads extravasated; scale bars, 500 and 50 μm (inset). Quantification of the area and capillary microbeads extravasated is shown on the bottom right; *n* = 3 to 5 mice for each group. **m**, Three-dimensional reconstruction of representative confocal images of whole-mount synovium from WT mice injected i.v. with OVA–AF647;rabbit polyclonal anti-OVA (RaOVA) ICs (40 μg of OVA–AF647 + 150 μg of RaOVA) 2 h before analysis. Arrowheads indicate sites where ICs extravasated; scale bars, 200 and 100 μm (*z*-stack images). **n**, Density map of a three-dimensional reconstruction of representative confocal images of whole-mount synovium from WT mice injected i.v. with microbeads or ICs. Data in **d**, **f** and **l** (right) were analyzed by two-tailed *t*-test. The center compartment was used as a control group in one-way analysis of variance (ANOVA) with a Dunnett’s test for **g**. Data in **i** and **l** (left) were analyzed by one-way ANOVA with Tukey’s post hoc test, and data in **b** were analyzed by two-tailed Wilcoxon rank-sum test. Data in **d**, **f**, **g**, **i** and **l** are shown as mean ± s.e.m. Images in **k**, **m** and **n** are representative of at least three independent experiments with similar results. Source data

**Extended Data Fig. 1:**
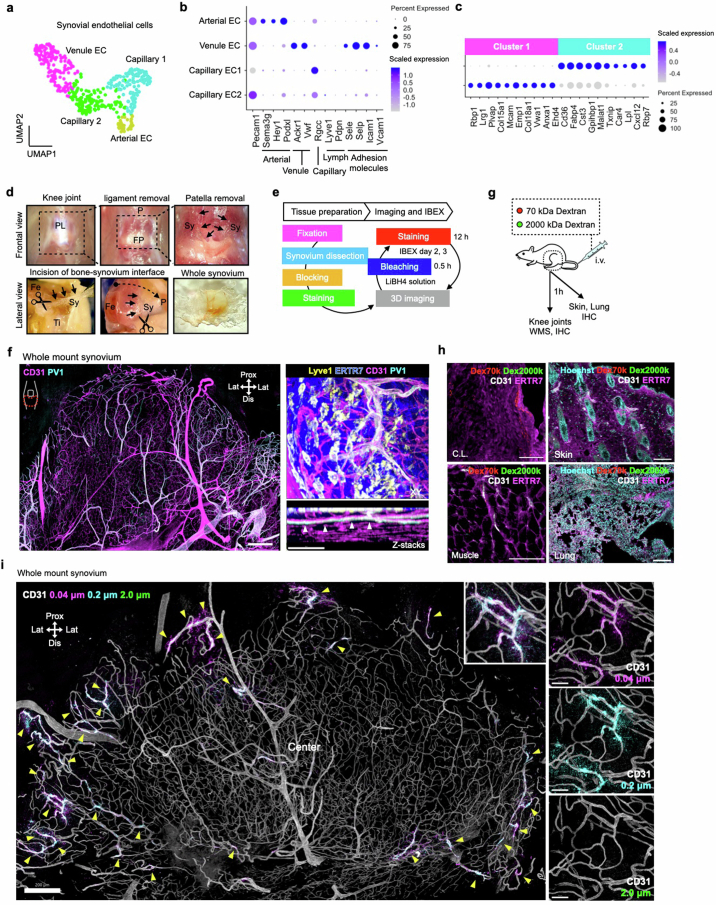
PV1+ capillaries with distinct molecular signatures localised at the L-SL interface of the synovium. (**a**) Uniform manifold approximation and projection (UMAP) visualizations of Pecam1+ endothelial cells in synovium. ScRNA-seq data from GSE145286. (**b**) Dot plots showing the scaled gene expression and percentage of cells expressing genes for cell markers and adhesion molecules in endothelial cell populations from scRNA-seq analysis. (**c**) Top 10 differentially expressed genes between two cell populations in synovial capillary endothelial cells. (**d**) Anatomic localization of the synovium from the frontal view and dissection protocol from the lateral view. P: patella, PL: patella ligament, FP: fat pad, Sy: synovium, Fe: femur, Ti: tibia. (**e**) Schematic depicting tissue preparation, imaging and iterative bleaching extends multiplexity (IBEX) protocol. (**f**) 3D reconstruction of representative confocal images of whole mount synovium. Arrowheads indicate PV1+ capillaries at the lining-sublining (L-SL) interface (Z-stack images). Bars, 200 and 50 μm. Images are representative of at least three independent experiments with similar results. (**g**) Schematic diagram showing the protocol. (**h**) Representative confocal images of sections of skin and lung from wild type mice injected i.v. with 70 and 2000 kDa Dextran (300 μg 70 kDa Dextran and 150 μg 2000 kDa Dextran) 1 h prior to analysis. Images are representative of at least three independent experiments with similar results. (**i**) 3D reconstruction of representative confocal images of whole mount synovium from wild type mice injected i.v. with fluorescently labeled microbeads of different sizes 1 h prior to analysis. Arrows indicate the sites where microbeads extravasated. Bars, 200 and 50 μm. Images are representative of at least three independent experiments with similar results.

To interrogate the synovial vasculature via which circulating stimuli gain access to the synovium, we analyzed endothelial cells from single-cell RNA-sequencing (scRNA-seq) data of mouse synovium^6^ (Extended Data Fig. 1a). Genes encoding adhesion molecules, such as *Sele*, *Selp* and *Icam1*, were mainly expressed on *Ackr1*^+^ postcapillary venules, where immune cells extravasate^19^ (Extended Data Fig. 1b). Capillary endothelial cells were also evident and, when considered in isolation, comprised two subsets (Fig. 1a). *Plvap* was exclusively expressed in cluster 1 and was among the top ten differentially expressed genes (DEGs) when comparing these two capillary cell clusters (Fig. 1b,c and Extended Data Fig. 1c). PV1 (encoded by *Plvap*) is a molecular component of fenestral diaphragms, conferring vascular permeability^20,21^, and its expression is indicative of fenestrated capillaries.

The synovium can be divided into a lining layer containing synovial fibroblasts, with overlying macrophages that interface with the synovial cavity, and a sublining layer with additional macrophage populations^7,22–24^. Sagittal section imaging showed that PV1^+^ capillaries were specifically located at the interface of the lining and sublining layers, termed hereafter as the lining–sublining (L–SL) interface (Fig. 1d). Although sagittal sections have been used as the gold standard to define the spatial organization of synovial cells, this method provides a limited snapshot of the synovial membrane as a whole, which stretches around the entire joint. Indeed, our knowledge of the global arrangement of synovial cells, particularly in homeostasis, is surprisingly rudimentary. To address these limitations, we developed a protocol to stereotactically dissect the entire synovium of knee joints (Fig. 1e and Extended Data Fig. 1d,e) and combined whole-mount synovial imaging with an iterative bleaching and staining protocol^25^ to enable multiparameter imaging. This revealed a dense PV1^–^ vascular network in the sublining layer and confirmed that PV1 was predominantly expressed on capillaries at the L–SL interface (Fig. 1f and Extended Data Fig. 1f). When considering the L–SL interface across the entire area of the synovium, we found that PV1^+^ capillaries were not uniformly distributed but rather were abundant at the periphery of the synovium in proximity to adjacent bones (Fig. 1g).

To test the functional importance of the distribution of PV1 capillaries, we administered 70-kDa and 2,000-kDa dextran intravenously (i.v.) and collected organs 1 h later. Although 70-kDa dextran highly extravasated at the diaphysial side of the growth plate in bone marrow, 2,000-kDa dextran was evident in the synovium (Fig. 1h,i and Extended Data Fig. 1g,h), predominantly in the L–SL interface (Fig. 1j,k). Similarly, i.v. administration of fluorescently labeled microbeads showed that 0.2-μm microbeads extravasated exclusively from PV1^+^ capillaries in the L–SL interface in the periphery of healthy synovium (Fig. 1l), whereas 2-μm microbeads were excluded (Extended Data Fig. 1i). Finally, we used a clinically relevant challenge; i.v. ICs (ovalbumin (OVA) opsonized with a polyclonal anti-OVA IgG^26^) also extravasated into the local synovial tissue from PV1^+^ capillaries (Fig. 1m,n). Together, these data indicate that circulating stimuli readily gain access to the healthy synovium via highly permeable PV1^+^ capillaries located at the L–SL interface in the periphery of the synovium.

### Three subsets of macrophages with distinct distribution patterns line synovial PV1^+^ capillaries

**Fig. 2:**
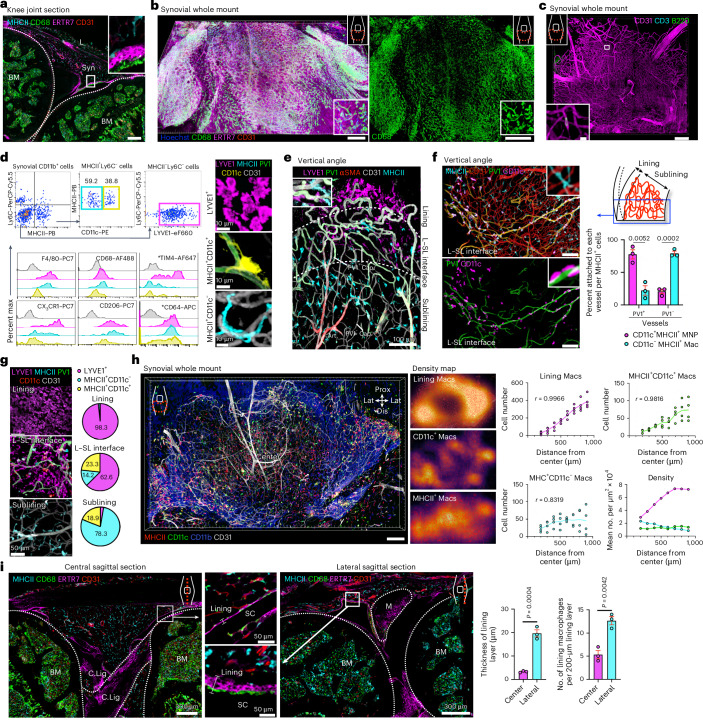
Three subsets of macrophages with distinct distribution patterns line synovial PV1^+^ capillaries. **a**, Representative confocal images of sections of healthy knee joints; BM, bone marrow; Syn, synovium; L, patella ligament; scale bar, 200 μm. **b**, Three-dimensional reconstruction of representative confocal images of whole-mount synovium; scale bars, 200 (left) and 300 μm (right). **c**, Three-dimensional reconstruction of representative confocal images of whole-mount synovium; scale bars, 200 and 10 μm. **d**, Gating strategy and flow cytometric analysis of three subsets of synovial macrophages with indicated cell surface markers. Shaded regions indicate staining with isotype controls. Data are representative of at least two independent experiments with similar results. The asterisks (*) indicate that fluorophores for LYVE1 were changed to apply the indicated antibodies. Representative confocal images of each subset of macrophages are shown on the right. **e**, Three-dimensional reconstruction of representative confocal images of whole-mount synovium from a vertical angle. The two dashed lines are (1) the border of lining layer and L–SL interface, and (2) L–SL interface and sublining layer. Cap., capillary; Art., arteriole. **f**, Three-dimensional reconstruction of representative confocal images of whole-mount synovium from a vertical angle; scale bars, 100 μm. Quantification of the percentage of CD11c^+^MHCII^+^ and CD11c^–^MHCII^+^ macrophages (Mac) attached to PV1^+^CD31^+^ or PV1^−^CD31^+^ vessels; *n* = 3 mice for each group. Data represent mean ± s.e.m. **g**, Three-dimensional reconstruction of representative confocal images at the indicated layers of whole-mount synovium. The pie graphs show the percentages of the three types of macrophages in the indicated layers of the synovium; *n* = 3 mice. **h**, Three-dimensional reconstruction of representative confocal images of whole-mount synovium (left) and density maps of three subsets of macrophages (middle); scale bar, 200 μm. The numbers and densities of each macrophage type in the indicated compartment of the synovium are shown on the right; *n* = 4 mice for each group. **i**, Representative confocal images of sections of healthy knee joints; M, meniscus; C.Lig, crescent ligament; SC, synovial cavity; *n* = 3 mice for each group. Data represent mean ± s.e.m. Data in **f** and **i** were analyzed by two-tailed *t*-test. Images in **a**–**e** are representative of at least two independent experiments with similar results. Source data

We next sought to interrogate which immune cells localized to these sites of potential vulnerability around PV1^+^ capillaries. ERTR7^+^ lining fibroblasts evident in sagittal sections (Fig. 2a) formed a uniform, tightly knit lining layer in the synovial whole-mount images (Fig. 2b), but CD68^+^ macrophages showed increased density in the periphery of the synovium (Fig. 2b and Extended Data Fig. 3a). No extravascular synovial T or B cells were detectable (Fig. 2c). We therefore focused on further characterizing the synovial macrophage populations.

**Extended Data Fig. 2:**
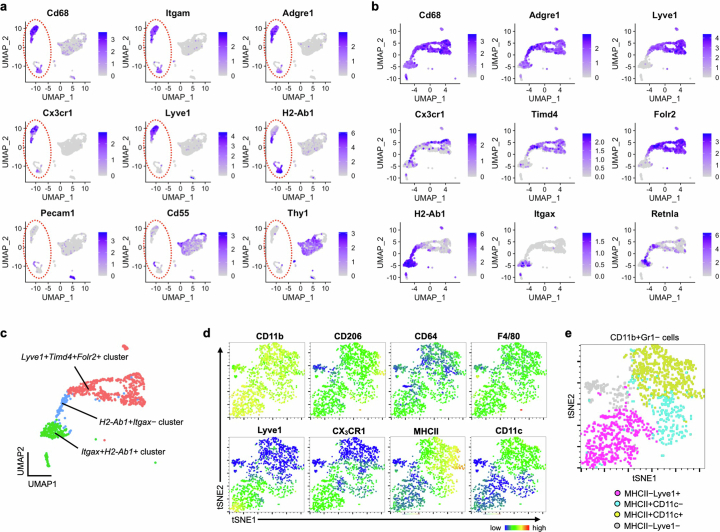
Gating strategy for healthy synovial macrophages. (**a**) UMAP visualisations of the healthy synovium and (**b, c**) myeloid cells (red circle) in the same dataset. ScRNA-seq data from GSE145286. (**d**, **e**) tSNE plot of surface marker expressions of CD11b + Gr1− cells in the healthy synovium using flow cytometry. Data from 6 mice were concatenated.

scRNA-seq data showed that synovial macrophages (*Cd68*^+^*Adgre1*^+^) can be divided into a *Cx3cr1*^+^ population (previously described as lining macrophages^7^), which also express *Lyve1*, *Timd4* and *Folr2*, an *H2-Ab1*^+^ population (previously described as interstitial macrophages^7^) and a subpopulation within the *H2-Ab1*^+^ population expressing *Itgax* (encoding CD11c; Extended Data Fig. 2a–c). Unsupervised assessment of myeloid surface markers using flow cytometry also showed that myeloid cells in the healthy synovium can be divided into major histocompatibility complex class II^+^CD11c^–^ (MHCII^+^CD11c^–^), MHCII^+^CD11c^+^ and MHCII^–^LYVE1^+^ clusters, which also express CX_3_CR1 (Extended Data Fig. 2d,e). These data informed the gating strategy subsequently used in our study characterizing three subsets of synovial macrophages, CD11c^+^IAIE(MHCII)^+^, CD11c^–^MHCII^+^ and MHCII^–^Ly6C^−^LYVE1^+^CX_3_CR1^+^cells, which were phenotypically and morphologically distinct (Fig. 2d). MHCII^–^LYVE1^+^ macrophages expressed CX_3_CR1 and TIM4, whereas CD206 and CD64 were expressed in all three populations.

To investigate the spatial distribution of macrophages relative to PV1^+^ capillaries, we performed whole-mount synovial imaging vertically at the edge of whole-mounted synovium. This showed that α-smooth muscle actin^+^ (αSMA^+^) arterioles give rise to PV1^+^ capillaries at the L–SL interface, which were surrounded by LYVE1^+^ macrophages (Fig. 2e). CD11c^–^MHCII^+^ macrophages lined both PV1^–^ and PV1^+^ vasculature, whereas CD11c^+^MHCII^+^ mononuclear phagocytes (MNPs) specifically localized to PV1^+^ vessels (Fig. 2f). Therefore, all three macrophage subsets were found in proximity to PV1^+^ capillaries in the L–SL interface (Fig. 2g). When considering the synovium as a whole, lining macrophages varied in their distribution density, with far fewer cells in the central region (Fig. 2h). In the sublining layer, CD11c^+^MHCII^+^ MNPs predominantly localized in the periphery, whereas CD11c^–^MHCII^+^ macrophages were uniformly distributed throughout the synovium (Fig. 2h). Sagittal sections confirmed that the density of lining macrophages and the thickness of the lining layer were significantly greater at the peripheral area near the meniscus between the femur and tibia than at the central area (Fig. 2i).

**Fig. 3:**
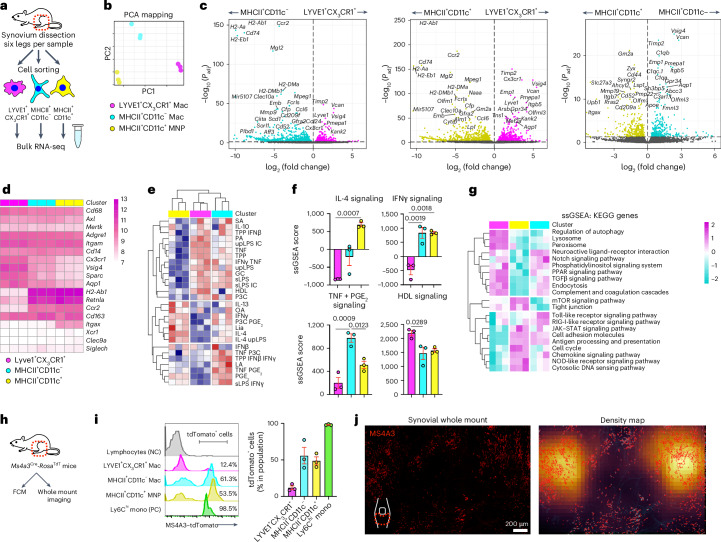
Three subsets of synovial macrophages show distinct transcriptomes and ontogenies. **a**, Illustration of the experimental protocol. **b**, Principal component analysis (PCA) of three subsets of synovial macrophages by RNA-seq; *n* = 3 mice for each plot and *n* = 9 mice for each population. **c**, Volcano plots showing DEGs between LYVE1^+^CX_3_CR1^+^, MHCII^+^CD11c^−^ and MHCII^+^CD11c^+^ macrophages from WT mice; *P*_adj_, adjusted *P* value. **d**, Heat map of the expression of canonical macrophage genes (normalized values) and dendritic cell markers in LYVE1^+^CX_3_CR1^+^, MHCII^+^CD11c^−^ and MHCII^+^CD11c^+^ macrophages from bulk RNA-seq analysis. **e**, Heat map of single-sample gene set enrichment analysis (ssGSEA) of three synovial macrophage subsets by RNA-seq. The signature genes from a previously published dataset (Xue et al.^27^) describing the transcriptional programs activated with 28 different stimuli were used; TPP, TNF+PGE_2_+P3C; IFN, interferon; PA, palmitic acid; LPS, lipopolysaccharide; TNF, tumor necrosis factor; GC, glucocorticoid; HDL, high-density lipoprotein; P3C, Pam3CysSerLys4; OA, oleic acid; Lia, linoleic acid; LA, lauric acid; sLPS, standard lipopolysaccharide; upLPS, ultrapure lipopolysaccharide. **f**, Quantification of ssGSEA scores for signaling pathways of the indicated stimuli for each subset; *n* = 3 mice for each plot and *n* = 9 mice for each population. Data represent mean ± s.e.m. **g**, Heat map of ssGSEAs of three synovial macrophage subsets with KEGG enrichment analysis (scaled normalized values). **h**, Illustration of the experimental protocol; FCM, flow cytometry. **i**, Flow cytometric analysis of MS4A3–tdTomato positivity of the indicated macrophage subsets from 10-week-old mice; *n* = 3 mice for each group. Data represent mean ± s.e.m.; NC, negative control; mono, monocytes; PC, positive control. **j**, Three-dimensional reconstruction of representative confocal images of whole-mount synovium and density map of MS4A3–tdTomato. Images are representative of three animals with similar results. Data in **f** were analyzed by one-way ANOVA with Tukey’s post hoc test, and data in **c** were analyzed by Wald test. Source data

Bulk RNA-seq of the sorted macrophage subsets showed that they were transcriptionally distinct, with LYVE1^+^CX_3_CR1^+^ macrophages expressing markers of lining macrophages (*Cx3cr1*, *Vsig4* and *Sparc*) and CD11c^+^MHCII^+^ and CD11c^–^MHCII^+^ macrophages demonstrating transcriptional similarity to interstitial macrophages (*H2-Ab1* and *Retnla*^7^; Fig. 3a–d). Compared to reference transcriptional signatures obtained from macrophages activated with different stimuli^27^, CD11c^+^MHCII^+^ MNPs were enriched for gene sets associated with M2 stimuli (interleukin-4 (IL-4) and IL-13), whereas LYVE1^+^CX_3_CR1^+^ macrophages were enriched for tumor necrosis factor (TNF) and high-density lipoprotein stimulation signatures (Fig. 3e,f). Pathway analysis showed that LYVE1^+^CX_3_CR1^+^ macrophages expressed genes related to endocytosis and the lysosome, whereas the MHCll^+^ subsets were enriched for gene sets related to cell adhesion and antigen processing and presentation (Fig. 3g).

**Extended Data Fig. 3:**
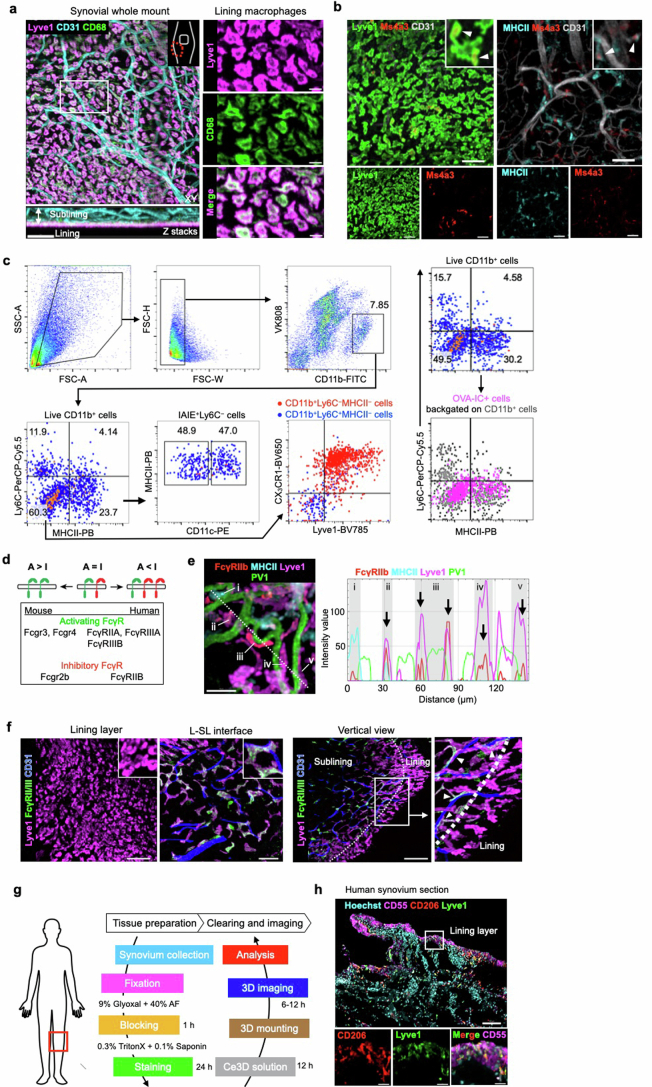
IC uptake and localisation of macrophages expressing FcγRII/lll in the synovium. (**a**) 3D reconstruction of representative confocal images of whole mount synovium. Bars, 50 (left) and 10 μm (right). Images are representative of at least three independent experiments with similar results. (**b**) 3D reconstruction of representative confocal images of whole mount synovium from Ms4a3^Cre^-Rosa^TdT^ mice. Bars, 50 μm. (**c**) Gating strategy and flow cytometric analysis of OVA-IC uptake by synovial macrophages from wild type mice injected i.v. with OVA-AF647;RaOVA (40 μg OVA-AF647 + 150 μg RaOVA) 2 h prior to analysis. Plots are representative of 3 mice. (**d**) Schematic of FcγR A:I ratios. (**e**) Representative plot profile intensity of cells (right) as indicated by the white line shown in the 3D reconstruction of representative confocal images of L-SL interface of whole mount synovium (left), showing five representative cells consisting of MHCII+ cells and Lyve1+ cells. Images are representative of at least two independent experiments with similar results. (**f**) 3D reconstruction of representative confocal images of indicated layer of whole mount synovium. Bars, 50 μm. Arrowheads indicate FcγRll/lll+ area at L-SL interface. (**g**) Schematic depicting tissue preparation, clearing, and imaging protocol of human synovium. (**h**) Representative confocal images of sections of human synovium. CD55+ area represents lining fibroblasts. Bars, 100 and 20 μm. Images are representative of at least two independent experiments with similar results.

We next investigated the kinetics of synovial macrophage replenishment by circulating monocytes using *Ms4a3*^Cre^*-Rosa*^TdT^ mice. *Ms4a3* is specifically expressed by granulocyte–monocyte progenitors, and this model efficiently fate maps monocytes and granulocytes but not lymphocytes or tissue dendritic cells^28^. In accordance with published reports showing that MHCII^+^ macrophages are replaced faster than MHCII^–^ macrophages in other organs^28^, in the synovium, almost half of MHCII^+^ macrophages were tdTomato^+^, whereas most of LYVE1^+^CX_3_CR1^+^ macrophages were tdTomato^–^ (Fig. 3h,i). An equivalent proportion of MHCII^+^CD11c^+^ MNPs were tdTomato^+^ compared to MHCII^+^CD11c^–^ macrophages, indicating that MHCII^+^CD11c^+^ MNPs have an ontogenic profile similar to macrophages rather than dendritic cells. Whole-mount synovial imaging of *Ms4a3*^Cre^*-Rosa*^TdT^ mice showed that tdTomato^+^ cells were predominantly distributed at the periphery of the synovium (Fig. 2j and Extended Data Fig. 3b).

### Synovial macrophages sample circulating ICs and present antigens

**Fig. 4:**
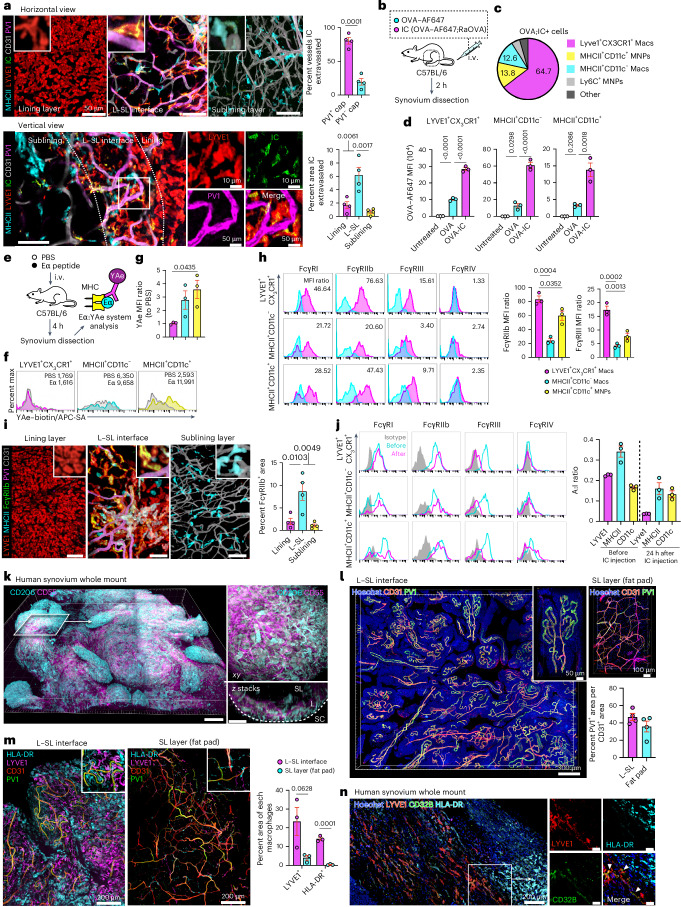
Synovial macrophages sample circulating ICs, and MHCII^+^ macrophages present antigens. **a**, Three-dimensional reconstruction of representative confocal images of the indicated layers and vertical views of whole-mount synovium from WT mice injected i.v. with OVA–AF647;RaOVA (40 μg of OVA–AF647 + 150 μg of RaOVA) 2 h before analysis. Quantification of the percentage of OVA-IC^+^ area within PV1^+^ and PV1^−^CD31^+^ area and the percentage of OVA-IC^+^ area in the lining layer, L–SL interface and sublining layer is shown on the right; *n* = 4 mice for each group. **b**, Schematic diagram showing the protocol. **c**, Pie graph showing the mean percentage of OVA-IC^+^ macrophage subsets among all OVA-IC^+^ cells; *n* = 3 mice. **d**, Scatter plots of mean fluorescence intensity (MFI) of OVA–AF647 and OVA–AF647;RaOVA; *n* = 3 mice for each group. **e**, Schematic diagram showing the protocol of antigen presentation in vivo using the Eα:YAe system. **f**,**g**, Flow cytometric analysis (**f**) and quantification (**g**) of YAe MFI of the indicated macrophage subsets from mice injected i.v. with Eα divided by that observed in macrophages from mice injected with PBS. Shaded regions indicate mice injected with PBS control; *n* = 3 mice for each group. **h**, Flow cytometric analysis of different types of synovial macrophages with indicated FcγRs. Cyan regions indicate staining with isotype controls. Scatter plots show the MFI ratio of each FcγR and isotype controls on each subset; *n* = 3 mice for each group. **i**, Three-dimensional reconstruction of representative confocal images of the indicated layers of whole-mount synovium; scale bars, 50 μm. Quantification of the percentage of FcγRllb^+^ area in the indicated layers is shown on the right; *n* = 4 mice for each group. **j**, Flow cytometric analysis of different types of synovial macrophages with the indicated FcγRs before and 24 h after IC injection. Gray regions indicate staining with isotype controls. The FcγR A:I ratios were calculated according to MFI ratios of activating (FcγRlll and FcγRlV) and inhibitory FcγRllb before and 24 h after IC injection on each subset; *n* = 3 mice for each group. **k**, Three-dimensional reconstruction of representative confocal images of whole-mount human synovium; scale bars, 100 (left) and 30 μm (right); L, lining layer; SL, sublining layer; SC, synovial cavity. **l**, Three-dimensional reconstruction of representative confocal images of whole-mount human synovium. Quantification of PV1^+^ area among CD31^+^ area in each layer; *n* = 3 individuals for each group. **m**, Three-dimensional reconstruction of representative confocal images of whole-mount human synovium. Quantification of LYVE1^+^ and HLA-DR^+^ area in the visual field is shown on the right; *n* = 3 individuals for each group. **n**, Three-dimensional reconstruction of representative confocal images of whole-mount human synovium; scale bars (right), 100 μm. Images are representative of at least two independent experiments with similar results. The arrowheads indicate the merged area for LYVE1 and CD32B. Data in **a** and **m** were analyzed by two-tailed *t-*test, and data in **d** and **g**–**i** were analyzed by one-way ANOVA with Tukey’s post hoc test. Data in **a**, **d**, **g**–**j**, **l** and **m** are shown as mean ± s.e.m. Source data

Given their proximity to PV1^+^ capillaries, we next investigated the capacity of synovial macrophages to take up circulating ICs in vivo. Two hours following i.v. administration, ICs extravasated from PV1^+^ capillaries at the L–SL interface (Fig. 4a,b), and uptake was evident mainly in LYVE1^+^CX_3_CR1^+^ macrophages, consistent with a previous report^24^. CD11c^+^MHCII^+^ and CD11c^–^MHCII^+^ macrophages were also capable of internalizing ICs, with a greater IC uptake than OVA alone (Fig. 4c,d and Extended Data Fig. 3c). Notably, CD11c^+^MHCII^+^ MNPs were also able to internalize and present circulating peptide (Eα; Fig. 4e–g), consistent with their enrichment for antigen processing and presentation gene sets (Fig. 3g).

Because Fcγ receptors (FcγRs) bind to the Fc portion of IgG to mediate the cellular effector responses to ICs, we analyzed their expression in synovial macrophages. Of note, the relative expression of activating receptors (FcγRIII/FcγRIV) and the inhibitory receptor (FcγRIIb) determines the activation threshold of a cell when encountering IgG ICs, termed the A:I ratio (Extended Data Fig. 3d)^29^. Flow cytometric analysis showed that LYVE1^+^CX_3_CR1^+^ macrophages expressed higher levels of FcγRIIb and FcγRIII than the other two subsets, but FcγRIV expression was absent in synovial macrophages (Fig. 4h). Whole-mount synovium imaging showed FcγRII/FcγRIII expression mostly at the L–SL interface on LYVE1^+^ macrophages (Fig. 4i and Extended Data Fig. 3e,f). Circulating ICs led to downregulation of FcγRIII expression in all three subsets, whereas the expression of the inhibitory receptor (FcγRIIb) was maintained (Fig. 4j), thereby increasing the activation threshold and preventing excessive (and potentially damaging) responses to circulating ICs.

To determine if this microarchitectural arrangement was present in human synovium, we optimized a whole-mount imaging system for human knee joint synovium using clearing-enhanced 3D (Ce3D), a tissue clearing solution (Extended Data Fig. 3g)^30^. Beneath the CD55^+^ lining layer fibroblasts^6^ (Fig. 4k and Extended Data Fig. 3h), an abundant PV1^+^ capillary network was present (Fig. 4l) and was tightly covered with LYVE1^+^ and HLA-DR^+^ macrophages and some LYVE1^+^ macrophages expressing CD32B (FCGR2B), whereas fat pad capillaries were scantily lined with perivascular macrophages (Fig. 4m,n), analogous to our observations in mouse synovium.

### Systemic IC challenge induced distinct responses in synovial macrophage subsets

**Fig. 5:**
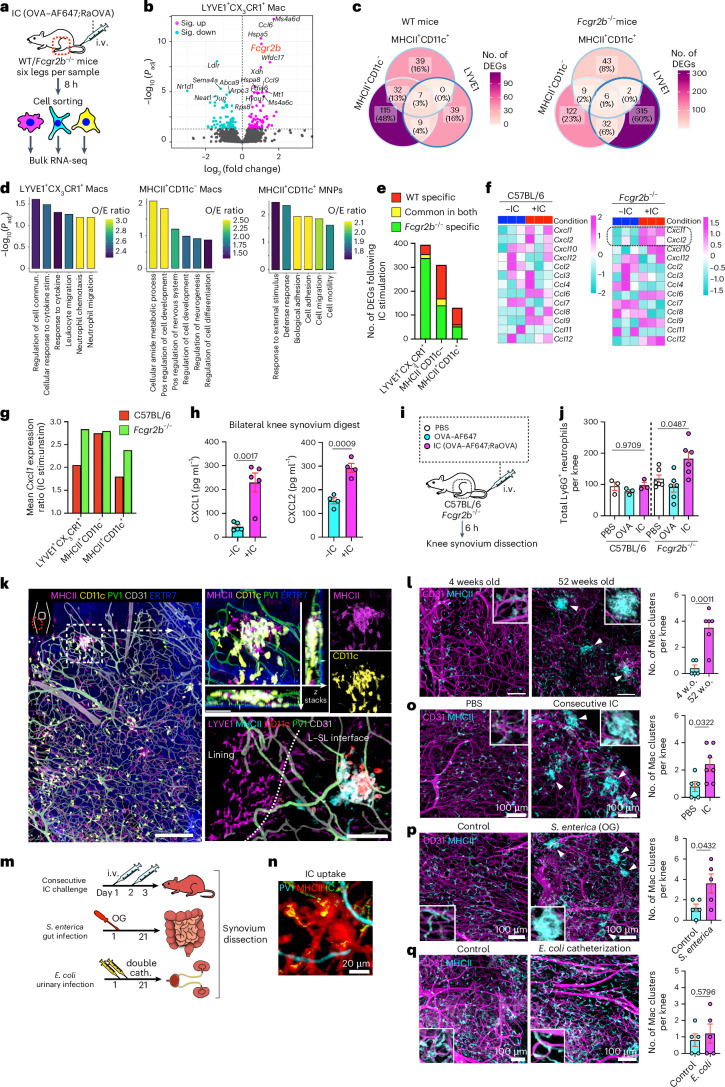
Systemic IC challenge induces distinct responses in synovial macrophage subsets. **a**, Schematic diagram showing the protocol for bulk RNA-seq. **b**, Volcano plot showing DEGs due to OVA-IC stimulation in LYVE1^+^CX_3_CR1^+^ macrophages from WT mice by RNA-seq; Sig., significantly. **c**, Venn diagram showing the number of common DEGs affected by IC stimulation between LYVE1^+^CX_3_CR1^+^, MHCII^+^CD11c^−^ and MHCII^+^CD11c^+^ macrophages in WT and *Fcgr2b*^−/−^ mice. **d**, Gene ontogeny (GO) analysis of DEGs specific to each macrophage type with all the DEGs of three macrophage subsets as the background gene list; commun., communication; stim., stimulation; Pos, positive; O/E, observed/expected. **e**, Number of DEGs in each synovial macrophage from WT and *Fcgr2b*^−/−^ mice and common DEGs in both strains. **f**, Heat map of the expression of chemokines (scaled normalized values) with or without IC injection in LYVE1^+^CX_3_CR1^+^ macrophages from WT and *Fcgr2b*^−/−^ mice. **g**, Ratio of mean *Cxcl1* expression in LYVE1^+^CX_3_CR1^+^, MHCII^+^CD11c^−^ and MHCII^+^CD11c^+^ macrophages from WT and *Fcgr2b*^−/−^ mice injected i.v. with or without ICs; stim/unstim, stimulated/unstimulated. **h**, CXCL1 and CXCL2 enzyme-linked immunosorbent assay (ELISA) of the synovial digestion from *Fcgr2b*^−/−^ mice with or without IC injection; *n* = 5 (CXCL1) and 4 (CXCL2) mice for each group. **i**, Schematic diagram showing the protocol. **j**, Flow cytometry quantification of synovial neutrophils (Ly6G^+^ gates) from WT and *Fcgr2b*^−/−^ mice injected i.v. with PBS, OVA or OVA;RaOVA 6 h before analysis; *n* = 3 (WT) and *n* = 6 (*Fcgr2b*^−/−^) mice. **k**, Three-dimensional reconstruction of representative confocal images of whole-mount synovium depicting an MHCII^+^ macrophage cluster around PV1^+^ capillaries; scale bars, 200 (left), 50 (top right) and 100 μm (bottom right). Images are representative of at least two independent experiments with similar results. **l**, Number of MHCII^+^ macrophage clusters with a diameter of >30 μm in the whole-mount synovium in 4- and 52-week-old mice; *n* = 5 (4-week-old) and 6 (52-week-old) mice; w.o., weeks old; scale bars, 100 μm. **m**, Schematic diagram showing the protocol of systemic challenges. **n**,**o**, Number and images of MHCII^+^ macrophage clusters with a diameter of >30 μm in the whole-mount synovium in mice injected i.v. with PBS or OVA-IC over 2 consecutive days and analyzed 24 h after the last injection; *n* = 5 and 7 mice for each group. **p**, Number of MHCII^+^ macrophage clusters in mice infected orally with PBS or 5 × 10^6^ *S. enterica* serovar Typhimurium and analyzed after 3 weeks; *n* = 5 mice for each group; OG, oral gavage. The arrowheads in **l**, **o** and **p** mark macrophage clusters. **q**, Number of MHCII^+^ macrophage clusters in mice inoculated with two doses of 4 × 10^7^ uropathogenic *E. coli* into the bladder and analyzed after 3 weeks; *n* = 5 mice for each group. Data in **h**, **l** and **o**–**q** were analyzed by two-tailed *t*-test, data in **j** were analyzed by one-way ANOVA with Tukey’s post hoc test, and data in **b** were analyzed by Wald test. Data are shown as mean ± s.e.m. in **h**, **j**, **l** and **n**–**q**. Source data

**Extended Data Fig. 4:**
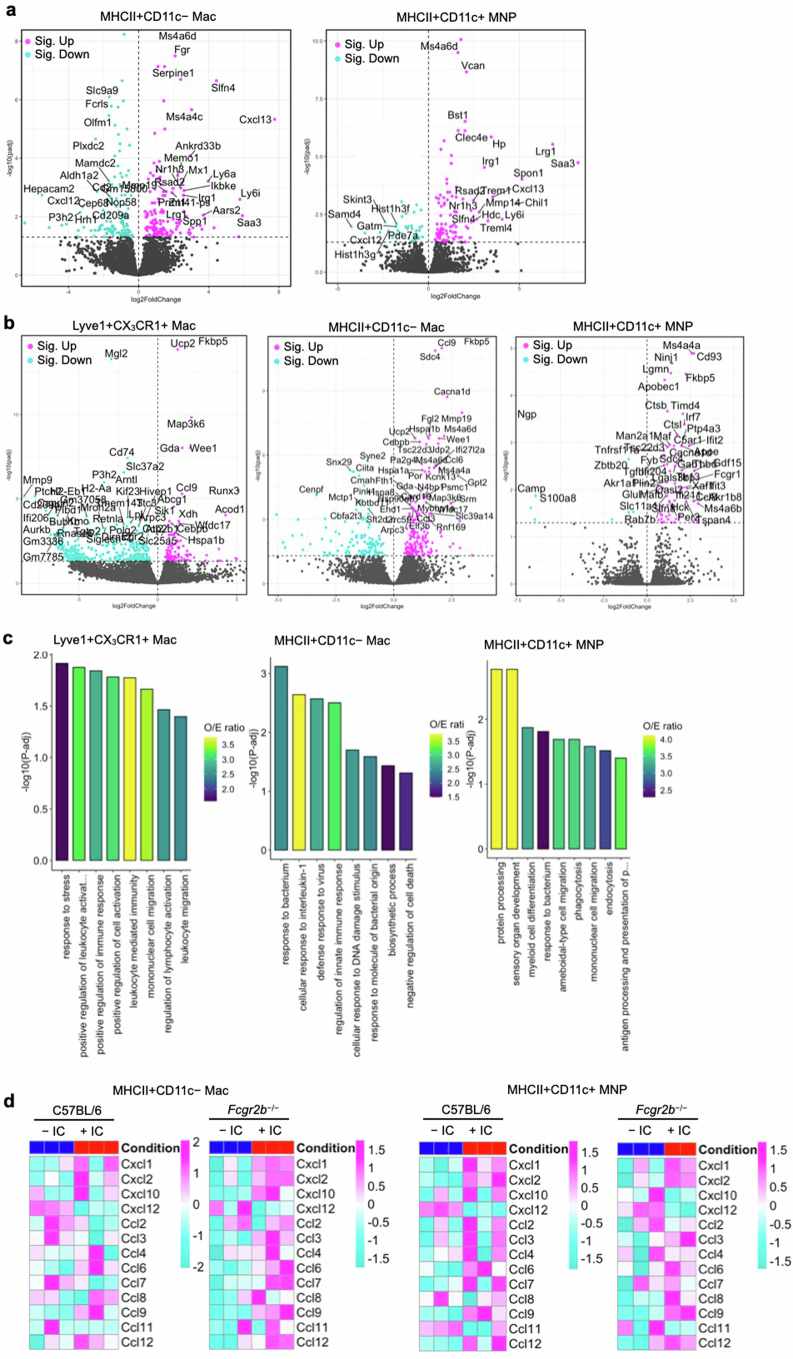
Depletion of FcγRllb alters the response to IC in each synovial macrophage subset. (**a**) Volcano plots showing DEGs due to OVA-IC stimulation in MHCII+CD11c− and MHCII+CD11c+ macrophages from wild type mice by RNA-seq. Wald test was used. (**b**) Volcano plots showing DEGs due to OVA-IC stimulation in Lyve1 + , MHCII+CD11c − , and MHCII+CD11c+ macrophages from Fcgr2b − /− mice by RNA-seq. Wald test was used. (**c**) Gene ontogeny (GO) analysis of DEGs specific to each macrophage subset with all the DEGs of 3 subsets as the background gene list. (**d**) Heat map of expression of chemokines with or without IV IC injection in MHCII+CD11c− and MHCII+CD11c+ macrophages from wild type and Fcgr2b − /− mice (scaled normalized values).

To further interrogate synovial macrophage responses to ICs, we performed bulk RNA-seq on sorted subsets following i.v. IC challenge in vivo in wild-type (WT) and FCGR2B-deficient (*Fcgr2b*^−/−^) mice (Fig. 5a). IC challenge was associated with increased *Fcgr2b* expression in LYVE1^+^CX_3_CR1^+^ macrophages in WT mice, consistent with a negative feedback loop (Fig. 5b). Less than 3% of DEGs were shared among the three macrophage subsets in both WT and FCGR2B-deficient mice, indicating subset-specific responses to circulating IC challenge (Fig. 5c). CD11c^+^MHCII^+^ and CD11c^–^MHCII^+^ macrophages shared more DEGs, including *Fcgr1*, *Mmp19*, *Irf7* and *Cxcl13*, than LYVE1^+^CX_3_CR1^+^ macrophages (Fig. 5c and Extended Data Fig. 4a). Pathway enrichment analysis using upregulated genes showed that LYVE1^+^CX_3_CR1^+^ macrophages were enriched with gene sets related to neutrophil migration, whereas CD11c^+^MHCII^+^ macrophages were enriched with gene sets related to cell adhesion and migration after IC stimulation (Fig. 5d).

FcγRIIb deficiency was associated with a greater increase in the number of DEGs in LYVE1^+^CX_3_CR1^+^ macrophages than in WT cells (Fig. 5e and Extended Data Fig. 4b), consistent with the high FcγRIIb expression detectable in this population. Because leukocyte activation- and neutrophil migration-associated genes were enriched in LYVE1^+^CX_3_CR1^+^ cells after i.v. IC challenge (Extended Data Fig. 4c), we further probed the expression of chemokines. We found a greater IC-induced upregulation of the neutrophil-recruiting chemokine *Cxcl1* in LYVE1^+^CX_3_CR1^+^ and CD11c^+^MHCII^+^ macrophages in *Fcgr2b*^−/−^ mice than in WT mice (Fig. 5f,g and Extended Data Fig. 4d), supporting the conclusion that FcγRllb is important for limiting neutrophil entry after IC challenge. We confirmed a significant increase in CXCL1 and CXCL2 protein expression and infiltrating neutrophils within the synovium of *Fcgr2b*^−/−^ mice following i.v. IC challenge (Fig. 5h–j).

Interestingly, given the enrichment of cell adhesion and migration gene sets in activated CD11c^+^MHCII^+^ macrophages, we occasionally noted clusters of MHCII^+^ macrophages tightly entwined around PV1^+^ capillaries in unchallenged synovium, with LYVE1^+^ macrophages absent from these aggregates (Fig. 5k). The number of these aggregates increased with age (Fig. 5l). We therefore hypothesized that they may arise in response to chronic circulating stimuli, including ICs, or those present in the context of inflammation/infection in distant organs. To test this, we challenged mice with i.v. IC twice over a period of 48 h (Fig. 5m). This resulted in the appearance of MHCII^+^ macrophage aggregates at 72 h (Fig. 5n,o). Furthermore, oral challenge with *Salmonella enterica* serovar Typhimurium, a colitogenic bacteria^31^, also induced synovial macrophage aggregates (Fig. 5p). However, chronic urinary tract infection with uropathogenic *Escherichia coli* did not induce macrophage aggregates in the synovium (Fig. 5q), suggesting that organ infection variably influences joints.

**Extended Data Fig. 5:**
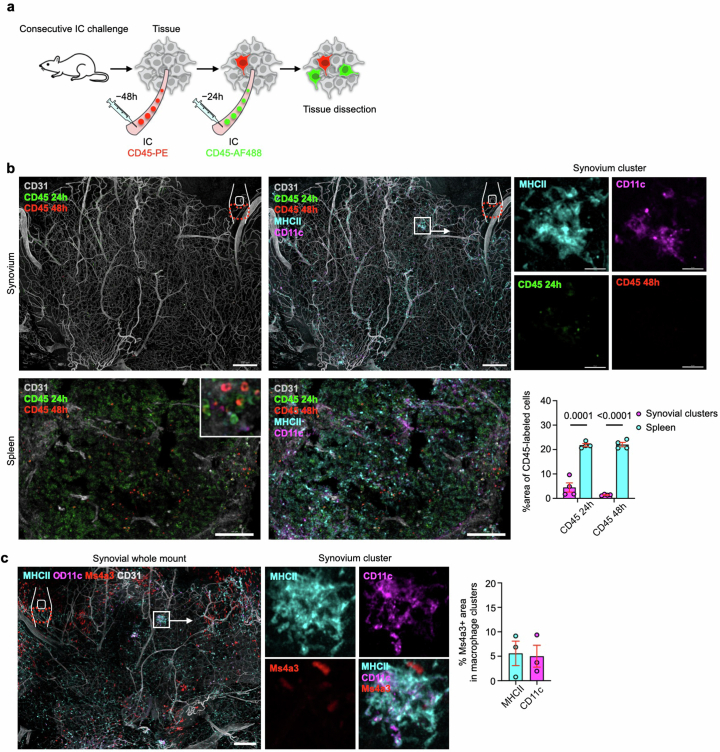
Monocyte contribution in the formation of MHCll+ macrophage clusters. (**a**) Schematic depicting the experiment. (**b**) 3D reconstruction of representative confocal images of whole mount synovium and sectional images of spleen after IC and anti-CD45 antibody injection. Bars, 200 (synovium) and 100 μm (spleen). Data represents mean ± SEM. Two-tailed t test was used. (**c**) 3D reconstruction of representative confocal images of whole mount synovium from Ms4a3^Cre^-Rosa^TdT^ mice. Bars, 200 μm. Data represents mean ± SEM. Source data

We next assessed the contribution of circulating monocytes to macrophage aggregate formation. A recent study^32^ established an in vivo protocol to fluorescently label intravascular leukocytes that were shielded from subsequent rounds of intravascular labeling once they entered tissues. We applied this system in our chronic IC stimulation model to assess the monocyte contribution to synovial macrophage aggregates. We administered i.v. ICs together with phycoerythrin (PE)-labeled anti-CD45 48 h before synovial collection, followed by administration of i.v. ICs and AF488-labeled anti-CD45 24 h before analysis (Extended Data Fig. 5a). Although we detected both 24- and 48-h time stamp signals in the spleen, synovial macrophage aggregates were not labeled with either 24- or 48-h time stamps (Extended Data Fig. 5b). Furthermore, using monocyte reporter *Ms4a3*^Cre^-*Rosa*^TdT^ mice, synovial macrophage aggregates were not labeled with tdTomato (Extended Data Fig. 5c), together indicating that these synovial macrophage clusters are derived from a local macrophage pool rather than circulating monocytes.

**Extended Data Fig. 6:**
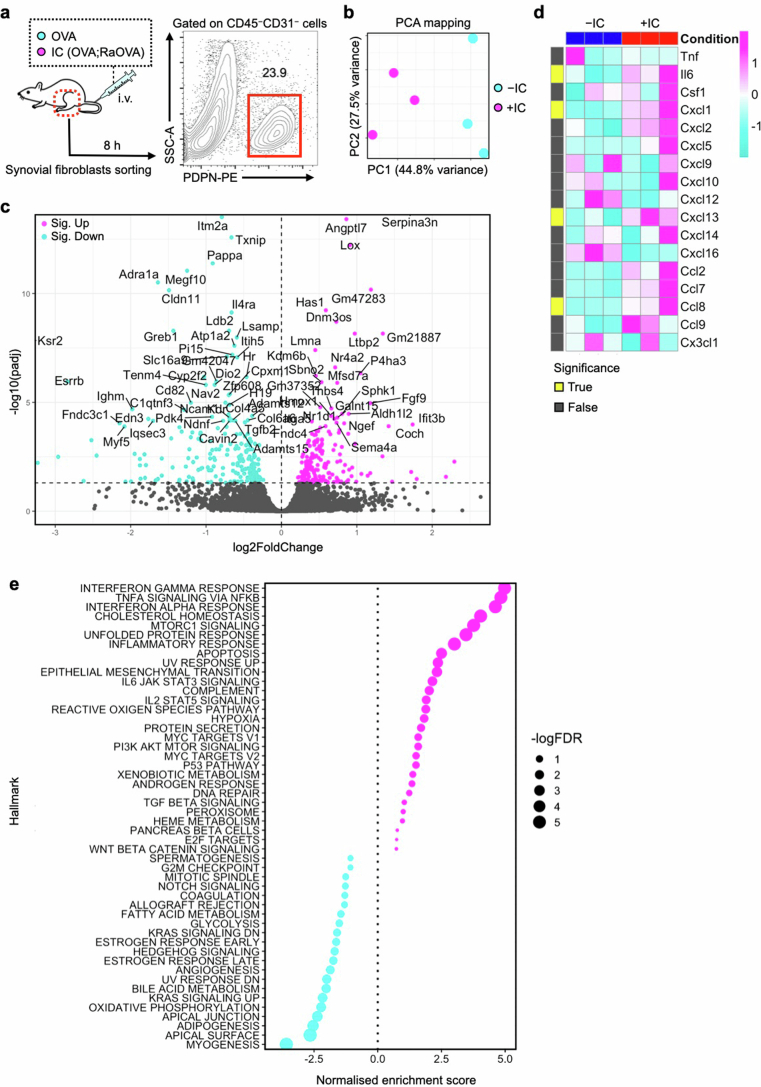
Transcriptomic changes of synovial fibroblast post systemic IC challenge. (**a**) Schematic depicting the protocol. (**b**) Principal component analysis of synovial fibroblasts by RNA-Seq. n = 3 mice for each population. (**c**) Volcano plot showing differentially expressed genes in synovial fibroblasts post IC challenge. Wald test was used. (**d**) Heatmap of inflammatory cytokines and chemokines of synovial fibroblasts by RNA-seq. (**e**) Gene set enrichment analysis of the bulk RNA-seq data.

We next asked whether fibroblasts play any role as secondary effectors, responding to cues produced by FcγR-expressing synovial macrophages after IC challenge (Extended Data Fig. 6a). Bulk RNA-seq of flow-sorted fibroblasts showed 417 IC-induced genes in synovial fibroblasts (Extended Data Fig. 6b,c), including *Cxcl1*, *Cxcl13* and *Ccl8* (Extended Data Fig. 6d). Enrichment analysis also showed that inflammatory response gene pathways, including interferon and IL-6–JAK–STAT3 signaling pathways, were upregulated following IC challenge (Extended Data Fig. 6e).

Together, these data show distinct responses of synovial macrophage subsets to circulating IC challenge, with LYVE1^+^CX_3_CR1^+^ macrophages poised to trigger neutrophil recruitment but held in check by FcγRllb expression. By contrast, CD11c^–^MHCII^+^ and CD11c^+^MHCII^+^ macrophages can present circulating antigens and respond to systemic immune stimuli, including ICs, by forming tight clusters around fenestrated capillaries, thus forming a physical barrier that might limit the spread of potentially harmful blood-borne cargo into the joint. In addition, synovial fibroblasts may function as secondary effectors, responding to macrophage cues.

### Synovial macrophages activate nociceptors with IL-1β and nociceptors reciprocally enhance macrophage responses through CGRP

**Fig. 6:**
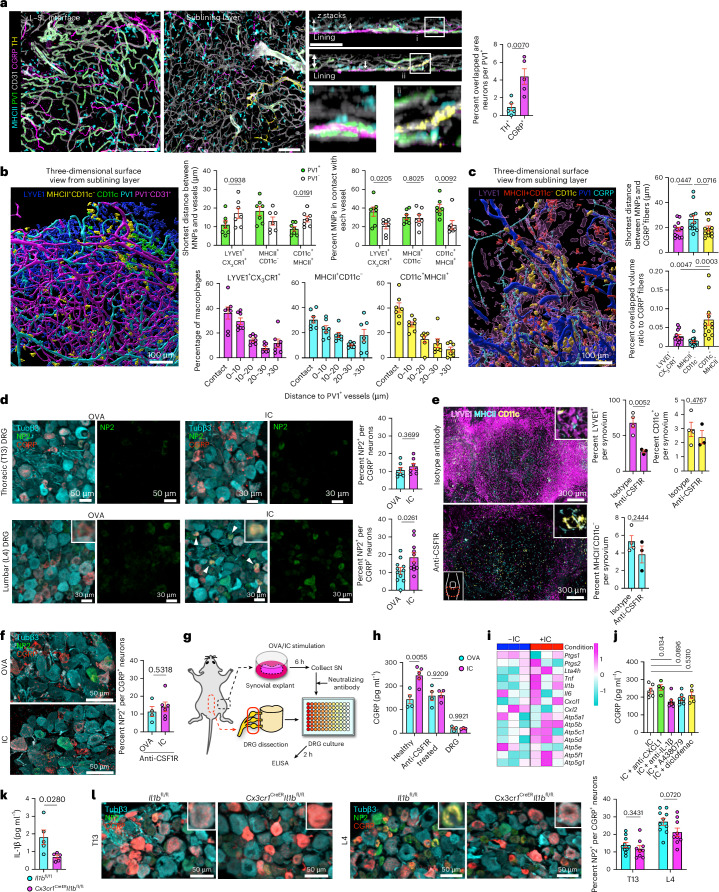
Synovial macrophages activate nociceptors in part through IL-1β. **a**, Three-dimensional reconstruction of representative confocal images of whole-mount synovium; scale bars, 100 μm. Arrows indicate CGRP^+^ neurons in *z*-stack images; *n* = 5 mice for each group. **b**, Three-dimensional reconstruction of representative confocal images of whole-mount synovium using the Surface module in Imaris and quantification of the distance between each macrophage subset and vessels; *n* = 7 mice for each group. Each plot indicates the mean value of each mouse. **c**, Three-dimensional reconstruction of representative confocal images of whole-mount synovium using the Surface module in Imaris and quantification of the distance or overlapped volume ratio between each macrophage subset and CGRP^+^ fibers; *n* = 12 mice for each group. Each plot indicates the mean value of each mouse. **d**, Representative confocal images of lumbar (L4) and thoracic (T13) DRG in mice injected i.v. with OVA or OVA-IC and analyzed after 6 h; *n* = 7 (T13) and 11 (L4) mice for each group; Tubβ3, tubulin-β3. The arrowheads indicate the merged area for NP2 and CGRP. **e**, Three-dimensional reconstruction of representative confocal images of whole-mount synovium from mice injected intraperitoneally (i.p.) with 400 μg of anti-CSF1R or isotype control antibody and analyzed after 72 h. Quantification of the percentage of area covered by each macrophage subset in the synovium is shown on the right; *n* = 4 (isotype) and 3 (anti-CSF1R) mice. **f**, Representative confocal images of L4 DRG in mice injected i.v. with OVA or OVA-IC 72 h after i.p. injection with 400 μg of anti-CSF1R; *n* = 4 (OVA) and 6 (IC) mice. **g**, Illustration of experimental protocol; SN, supernatant. **h**, CGRP ELISA of DRG culture SN stimulated with SN from OVA- or IC-stimulated synovial explants from indicated mice or directly stimulated with OVA or IC; *n* = 3 to 5 mice for each group. **i**, Heat map of the expression of potential candidates responsible for immune-driven pain in bulk RNA-seq data from IC-stimulated LYVE1^+^CX_3_CR1^+^ macrophages (scaled normalized values). **j**, CGRP ELISA of DRG culture supernatants stimulated with supernatants from IC-stimulated synovial explants. Indicated neutralizing antibodies (4 μg ml^–1^) or A438079 (100 μM) was added to synovial SN before adding to DRG neurons. Diflofenac (200 μM) was added with IC when stimulating synovial explants; *n* = 6 (IC), 4 (CXCL1), 7 (IL-1β), 7 (A438079) and 5 (Coxi) mice. **k**, IL-1β concentration of the synovial digestion measured by cytometric bead array from *Il1b*^fl/fl^ and *Cx3cr1*^CreER^*Il1b*^fl/fl^ mice 12 h after i.p. injection of 2 mg per kg (body weight) lipopolysaccharide (LPS); *n* = 5 mice for each group. **l**, Representative confocal images of T13 and L4 DRG in *Il1b*^fl/fl^ and *Cx3cr1*^CreER^*Il1b*^fl/fl^ mice injected i.v. with OVA-IC. Mice were i.p. injected with tamoxifen twice 48 h apart 2 weeks before IC injection and analyzed 6 h after IC injection; *n* = 10 (*Il1b*^fl/fl^) and 9 (*Cx3cr1*^CreER^*Il1b*^fl/fl^) mice. Data in **a**, **b**, **d**–**f**, **h**, **k** and **l** were analyzed by two-tailed *t*-test. The IC group was used as a control group in a one-way ANOVA with Dunnett’s post hoc test in **j**. Data in **c** were analyzed by one-way ANOVA with Tukey’s post hoc test. Data in **a**–**f**, **h** and **j**–**l** represent mean ± s.e.m. Source data

**Extended Data Fig. 7:**
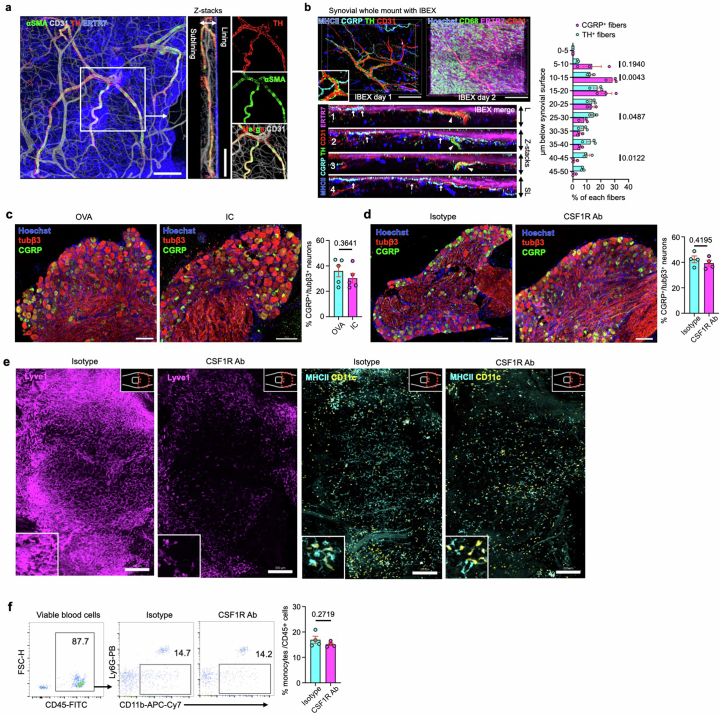
Distribution of TH^+^ and CGRP^+^ fibres in the synovium using IBEX. (**a**) 3D reconstruction of representative confocal images of whole mount synovium stained for tyrosine hydroxylase (TH). Bars, 100 μm. Images are representative of at least three independent experiments with similar results. (**b**) 3D reconstruction of representative confocal images of iterative bleaching extends multiplexity (IBEX) of whole mount synovium. Bars, 100 μm. Four Z-stack images of IBEX of whole mount synovium (bottom). Quantification of the penetration depth of CGRP^+^ and TH^+^ fibres as a percentage among total fibres for each subset (right). n = 3 mice for each group. (**c**) Representative confocal images of L4 dorsal root ganglia (DRG) from mice intravenously injected with OVA or IC. Bars, 100 μm. n = 5 mice for each group. (**d**) Representative confocal images of L4 DRG from mice intraperitoneally injected with isotype control or anti-CSF1R antibody. Bars, 100 μm. n = 4 mice for each group. (**e**) Enlarged images for each macrophage subset in Fig. 6e. (**f**) Flow cytometry plot showing the number of monocytes from mice intraperitoneally injected with isotype control or anti-CSF1R antibody 72 h prior to assessment. Data represents mean ± SEM and two-tailed t test was used for in **b**, **c**, **d**, **f**. Source data

**Extended Data Fig. 8:**
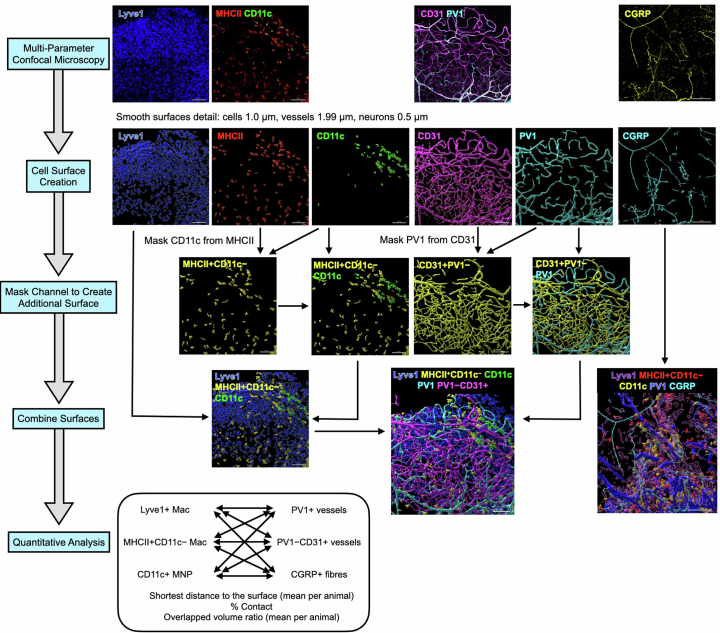
Protocol for quantifying spatial distribution of three types of macrophages, vessels, and CGRP^+^ fibres. Multi-parameter imaging of whole mount synovium was reconstructed with each fluorescent staining and quantitative analysis was performed.

As joint pain is one of the most common features of systemic challenges, we next sought to determine the anatomical relationship between PV1^+^ capillaries and neurons. Sympathetic tyrosine hydroxylase^+^ (TH^+^) neurons primarily colocalized with αSMA^+^ arterioles in the sublining layer (Extended Data Fig. 7a), whereas calcitonin gene-related peptide^+^ (CGRP^+^) nociceptor neuronal fibers branched into the L–SL interface around PV1^+^ capillaries (Fig. 6a and Extended Data Fig. 7b). Quantification of the spatial location of macrophages, PV1^+^ vessels, PV1^–^CD31^+^ vessels and CGRP^+^ fibers (Extended Data Fig. 8) indicated that approximately 30–40% of each macrophage subset were in direct contact with PV1^+^ vessels with LYVE1^+^CX_3_CR1^+^ macrophages, and CD11c^+^ MNPs preferentially localized to PV1^+^ vessels compared to PV1^–^ vessels (Fig. 6b). The average distance between CGRP^+^ fibers and macrophages was within 30 μm, and CD11c^+^ MNPs had the highest overlapped volume with CGRP^+^ fibers (Fig. 6c).

**Extended Data Fig. 9:**
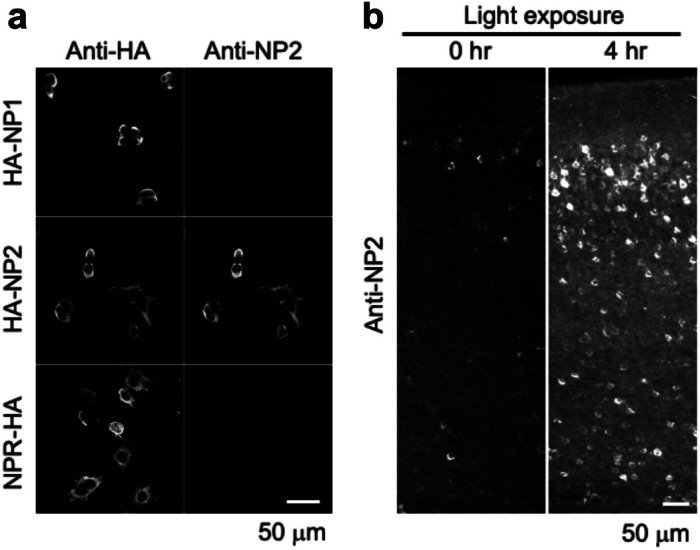
Validation of anti-NP2 antibody. (**a**) Immunocytochemical analysis of HA-tagged NP1, NP2 and NPR (full-length) expressed in HEK293 cells. Newly raised anti-NP2 antibody showed selective signals for NP2. (**b**) Specific immunostaining of endogenous NP2 in the visual cortex after the light stimulation. Anti-NP2 antibody detected the increase of NP2 protein expression in all layers of visual cortex 4 hours after light exposure compared with 0 hr exposure control. Bars, 50 μm.

CGRP^+^ neuronal cell bodies were evident in the L4 dorsal root ganglia (DRG) that innervates the knee joint^33^ (Extended Data Fig. 7c). Systemic IC injection induced activation of CGRP^+^ nociceptors in L4 but not T13 DRGs, as evidenced by neuronal pentraxin 2 (NP2) expression (Fig. 6d), an activity-dependent immediate early gene product^34^ (Extended Data Fig. 9) that facilitates excitatory responses in peptidergic DRG neurons, including CGRP^+^ afferents^35^. The proportion of CGRP^+^ neuronal bodies in the L4 DRGs did not change with IC injection nor anti-CSF1R treatment (Extended Data Fig. 7c,d).

**Extended Data Fig. 10:**
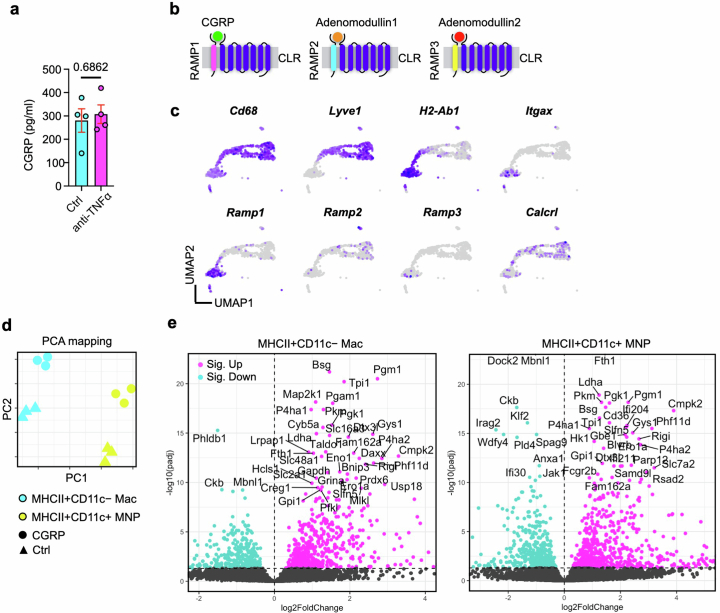
CGRP induces distinct transcriptional changes in MHCII+CD11c− and MHCII+CD11c+ macrophages. (**a**) CGRP ELISA of DRG culture supernatants stimulated with supernatants from IC stimulated synovial explant. TNF neutralizing antibodies (4 μg/ ml) or isotype control were added to synovial supernatants before adding to DRG neurons. n = 4 mice for each group. Data represents mean ± SEM. Two-tailed t test was used. (**b**) Illustration of receptor activity-modifying protein (RAMP) association on calcitonin receptor-like receptor (CLR). (**c**) Expression of marker genes for macrophages and neuropeptide receptors in healthy synovium. scRNA-seq data from GSE145286. (**d**) Principle component analysis (PCA) of MHCII+CD11c− and MHCII+CD11c+ macrophages from synovial explant of WT mice treated with 100 nM CGRP or control for 4 hours by RNA-seq. n = 3 mice were compiled for each plot and n = 18 mice for each population. (**e**) Volcano plots showing differentially expressed genes in MHCII+CD11c− and MHCII+CD11c+ macrophages with CGRP stimulation. Wald test was used. Source data

Because neurons do not express FcγRs and are therefore incapable of direct detection of circulating ICs, we hypothesized that adjacent FcγR-expressing macrophages relay their presence and activate CGRP^+^ nociceptors. To test this, we used anti-CSF1R that preferentially depleted LYVE1^+^ macrophages (Fig. 6e and Extended Data Fig. 7e,f). This attenuated the activation of CGRP^+^ nociceptors (Fig. 6f), confirming potential cross-talk between macrophages and CGRP^+^ nociceptors. Because anti-CSF1R potentially depletes macrophages in other tissues, we added supernatants from synovial whole-mount explants stimulated with OVA or IC to DRG neurons to validate a specific effect of synovial macrophages ex vivo (Fig. 6g). This induced the release of CGRP from DRG neurons, an effect absent in IC-stimulated synovium dissected from anti-CSF1R-treated mice (Fig. 6h). We next used our bulk RNA-seq data from IC-stimulated LYVE1^+^CX_3_CR1^+^ macrophages to prioritize inhibitors for testing in our ex vivo DRG culture model. This analysis identified several candidate molecules upregulated in IC-stimulated macrophages that have previously been described to mediate immune cell interactions with neurons^17,36^, including *Cxcl1*, *Tnf* and *Il1β* (Fig. 6i), and neutralization of IL-1β partly suppressed the excitation of DRG neurons (Fig. 6j and Extended Data Fig. 10a). Because *Cx3cr1*^CreER^ mice label lining macrophages in naive animals^7,24^, we crossed *Cx3cr1*^CreER^ mice with *Il-1β*^fl/fl^ mice to ablate IL-1β in LYVE1^+^CX_3_CR1^+^ macrophages. Cre recombinase activation of *Cx3cr1*^CreER^*Il-1β*^fl/fl^ mice resulted in a marked reduction of IL-1β production in the synovium (Fig. 6k), and we observed a reduction in nociceptor activation in L4 DRGs but not in nonjoint innervating T13 DRGs, demonstrating a role for synovial lining macrophage-derived IL-1β in activating nociceptor neurons (Fig. 6l).

**Fig. 7:**
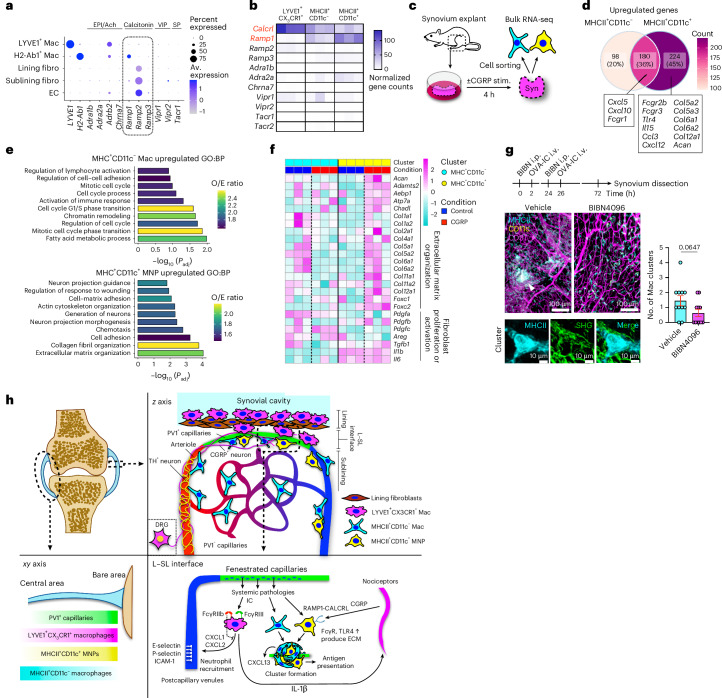
Nociceptors reciprocally enhance synovial macrophage responses through CGRP. **a**, Dot plots showing the average expression levels and the percentage of cells from each cluster expressing genes for neuropeptide receptors. scRNA-seq data are from GSE145286; EPI, epinephrine; Ach, acetylcholine; VIP, vasoactive intestinal polypeptide; SP, substance P; EC, endothelial cell; fibro, fibroblast; Av., average. **b**, Heat map of expression of genes for neuropeptide receptors in LYVE1^+^CX_3_CR1^+^, MHCII^+^CD11c^−^ and MHCII^+^CD11c^+^ macrophages from bulk RNA-seq analysis (normalized values). *Ramp1* and *Calcrl*, which encode proteins that form the CGRP receptor complex, are highlighted in red font. **c**, Illustration of the experimental protocol for bulk RNA-seq analysis. **d**, Venn diagram showing the number of distinct and common DEGs affected by CGRP stimulation between MHCII^+^CD11c^−^ and MHCII^+^CD11c^+^ macrophages. **e**, Gene ontogeny (GO) analysis of DEGs specific to each macrophage type with all the DEGs of two macrophage subsets as the background gene list. Fisher’s exact test was used; BP, biological process. **f**, Heat map of expression of genes related to extracellular matrix organization and fibroblast proliferation with CGRP stimulation in MHCII^+^CD11c^−^ and MHCII^+^CD11c^+^ macrophages (scaled normalized values). The vertical dashed lines mark the border of control and CGRP groups. **g**, Number of MHCII^+^ macrophage clusters with a diameter of >30 μm and second harmonic generation (SHG) on the clusters in the whole-mount synovium in mice injected i.v. with OVA-IC over 2 consecutive days after treatment with BIBN4096 (BIBN; 500 μg per kg (body weight) i.p.) or vehicle; *n* = 11 mice for each group. Data are shown as mean ± s.e.m. and were analyzed by two-tailed *t*-test. **h**, Schematic diagram of the immune-histochemical microarchitecture of the entire synovium and the sentinel unit surrounding PV1^+^ capillaries; ECM, extracellular matrix. Source data

Finally, we asked whether cross-talk between immune cells and nociceptor neurons in the synovium may also involve reciprocal signals from neurons to macrophages. scRNA-seq data of healthy synovium^6^ showed that *Ramp1*, *Ramp2* and *Calcrl* were the most highly expressed among neuropeptide receptor transcripts and *Ramp1*, which forms a complex with calcitonin receptor-like receptor to form the CGRP receptor, was particularly expressed in MHCII^+^ macrophages (Fig. 7a and Extended Data Fig. 10b,c). Bulk RNA-seq data confirmed *Ramp1* expression in MHCII^+^ macrophages, particularly the CD11c^+^ population (Fig. 7b). To investigate the functional importance of CGRP receptor expression in MHCII^+^ synovial macrophages, we stimulated whole synovial explants with CGRP for 4 h and sorted both MHCII^+^ macrophage subsets for bulk RNA-seq (Fig. 7c). We observed robust transcriptional responses to CGRP stimulation (Extended Data Fig. 10d), with some genes increased in both subsets, but almost half of the DEGs were unique to each macrophage subset (Fig. 7d and Extended Data Fig. 10e). In CD11c^+^MHCII^+^ MNPs, CGRP induced upregulation of *Fcgr2b*, *Fcgr3* and *Tlr4*, and gene sets related to cell adhesion and extracellular matrix organization, including collagen genes (Fig. 7d–f). We next treated mice with BIBN4096, an antagonist of RAMP1 signaling. Pretreatment with BIBN4096 partially suppressed MHCII^+^ macrophage cluster formation and collagen deposition, the latter visualized by second harmonic generation around the macrophage clusters induced by consecutive i.v. IC challenges (Fig. 7g). Together, these data show that following i.v. IC challenge, LYVE1^+^CX_3_CR1^+^ macrophages in the synovium activate nociceptors with IL-1β, and, in turn, nociceptors enhance CD11c^+^MHCII^+^ macrophage responses to IC through CGRP to coordinate a synovial defense system against circulating challenges (Fig. 7h).

## Discussion

Joint pain or inflammation is a common and early feature of several systemic diseases. These include autoimmune diseases, such as systemic lupus erythematosus, adult onset Still’s disease and inflammatory bowel disease, as well as infection in organs distant to the musculoskeletal system, including enteric or genitourinary infections, streptococcal pharyngitis and viral infections, for example, parvovirus^2–4^. The pathogenic mediators vary, but circulating ICs or microbial antigens have been implicated^37^. However, why joints are highly responsive to systemic inflammation is unknown. We sought to address this question, developing a whole-mount imaging system of the entire synovium to profile the vascular, neuronal and immune components. This revealed that highly permeable PV1^+^ capillaries were specifically located at the L–SL interface in the periphery of the synovium, enabling entry of circulating stimuli into the joint. We found that this area of vulnerability was occupied by three subsets of macrophages that demonstrated distinct responses to systemic IC challenge and interacted with nociceptor neurons, forming a blood–joint barrier (BJB) to defend joint tissue.

The location of fenestrated capillaries and their defensive macrophage–nociceptor sentinel unit in the periphery of the synovium at the synovia–bone interface is particularly interesting because this is the site of onset and formation of destructive granulomatous pannus in RA^13,15^. RA is characterized by circulating antibodies, including rheumatoid factor (IgM antibodies specific for the Fc portion of IgG) and anti-citrullinated protein IgG^1^. Our study identifies a fenestrated capillary bed that would enable access of autoantibodies or IC to the synovium at the very site of pannus formation. We found that adjacent macrophages expressed FcγRs, enabling responses to circulating IC and autoantibodies that might access the synovium via the fenestrated vasculature. Indeed, the inhibitory receptor FcγRIIb was a critical regulator of IC-induced activation of LYVE1^+^CX_3_CR1^+^ macrophages, with neutrophil recruitment to the synovium evident in its absence. Our data are consistent with human studies in RA showing that individuals with an *FCGR2B* polymorphism associated with receptor dysfunction demonstrate increased joint damage^38^.

The presence of highly permeable PV1^+^ capillaries in the synovium provides a mechanistic explanation for the joint manifestations of infections affecting organs distal to the musculoskeletal system. Previous studies have identified the presence of bacterial rRNA in a wide spectrum of inflammatory joints^39^ and viral nucleic acids in viral-associated arthritis^40^. Together, this suggests that the synovium is accessible to circulating microbes or microbial components. Clinically, reactive arthritis is characterized by a neutrophil-rich synovial effusion^41^, consistent with our observation that PV1^+^ capillary-associated synovial macrophages avidly produce neutrophil-recruiting chemokines in response to circulating stimuli. In addition, we found that *S. enterica* serovar Typhimurium colitis promoted macrophage cluster formation around PV1^+^ capillaries in the synovium, potentially reflecting the pathology of reactive arthritis.

Using the *Ms4a3*^Cre^-*Rosa*^TdT^ model, we assessed the contribution of circulating monocytes to synovial macrophages under steady-state conditions. Compared to other organs that have no monocyte contribution (microglia, Kupffer cells and Langerhans cells), fast replacement (gut and dermis) or slow replacement (kidney and spleen)^28^, synovial macrophages have heterogeneous kinetics, with the MHCII^+^ macrophage pool having a greater monocyte contribution than LYVE1^+^CX_3_CR1^+^ lining macrophages. Whole-mount synovial imaging also showed that monocytes preferentially replace synovial macrophages in the synovia–bone interface where proliferative pannus arises in RA, which further highlights the importance of PV1^+^ fenestrated capillaries as sites where joint pathology is initiated.

The activation of nociceptors alerts humans to the presence of harmful stimuli, leading to pain^42^. There has been recent interest in potential interactions between nociceptors and immune cells, including via neuropeptides, mainly in the context of infectious diseases where bacteria or fungi can directly activate nociceptors^16,43–45^. By contrast, our study shows that synovial macrophages sense local IC challenge and stimulate nociceptors, an effect mediated in part through IL-1β. IL-1β has also been previously implicated in augmenting pain sensitivity by promoting prostaglandin production^46^, upregulating transient receptor potential cation channel subfamily V member 1 (ref. ^47^) and generating action potentials in a p38 MAP kinase-dependent manner^48^. Our results suggest that IL-1 receptor antagonists may be a useful therapy for joint pain in a variety of IC-mediated diseases. Other candidates responsible for immune-driven joint pain should be explored in future studies. Furthermore, we found that this immune-driven nociceptor activation led to CGRP production, which in turn affected CD11c^+^MHCII^+^ MNPs, increasing the expression of IC and pathogen-associated molecular pattern receptors, such as *Fcgr2b*, *Fcgr3* and *Tlr4*. This heightened ability to detect and be activated by circulating stimuli reinforces a physical barrier to defend the area of vulnerability around PV1^+^ capillaries. Among the interstitial macrophages, CD11c^–^MHCII^+^ macrophages and CD11c^+^MHCII^+^ MNPs showed distinct spatial localization (Fig. 2f), transcriptional profiles in the homeostatic state (Fig. 3b) and responses to IC and CGRP stimulation (Figs. 5c and 7d–f).

Finally, perhaps the most interesting question is why a BJB equipped to sense circulating stimuli and telegraph their presence via pain has evolved. It is tempting to speculate that there might be a selective advantage to such a warning system at both an individual and species level. First, arthralgia might reduce mobility and thereby preserve energy for immune responses. Second, reduced movement may limit the transmission of infectious diseases to other individuals. Similar concepts have been proposed to explain why some infection-associated cytokines lead to increased anxiety and reduced socialization^49^.

Together, our data show how the unique anatomical arrangement of cells in the peripheral region of the synovium provides access to circulating immune stimuli through fenestrated capillaries and how this is policed by interacting macrophages and nociceptor neurons. Our findings have implications for the development of treatment strategies for immune-driven arthralgia.

## Methods

### Mice

WT mice (C57BL/6J background) were bred in-house or purchased from The Jackson Laboratory. Transgenic mice expressing Venus enhanced yellow fluorescent protein under the control of the *Itgax* promoter were a gift from M. Nussenzweig (Rockefeller University). *Fcgr2b*^*−/−*^ mice were kindly provided by J. Ravetch (Rockefeller University) and S. Bolland (US National Institutes of Health and US National Institute of Allergy and Infectious Diseases)^50^. *Il1b*^fl/fl^ floxed mice were crossed with tamoxifen-inducible B6.129P2(C)-*Cx3cr1*^*tm2.1(cre/ERT2)Jung*/J^ mice (The Jackson Laboratory, 020940) as described before^51^. Cre recombinase activity was induced by two i.p. injections of tamoxifen (2 mg 100 μl^–1^, Sigma-Aldrich, dissolved in corn oil) 48 h apart in 5- to 7-week-old male mice 2 weeks before the experiment.

In all experiments, both male and female mice were used. For all in vivo experiments, 8- to 20-week-old mice were used unless otherwise mentioned. Mice were maintained under specific pathogen-free conditions at a Home Office-approved facility with controlled humidity and temperature with a 12-h light/12-h dark cycle in the United Kingdom. All procedures were ethically approved by the University of Cambridge Animal Welfare and Ethical Review Body and were performed in accordance with the United Kingdom Animals (Scientific Procedures) Act of 1986 under the authority of a UK Home Office Licence.

### Human samples

Human synovial specimens were obtained from individuals with osteoarthritis undergoing replacement surgery or synovectomy with prior ethical approval (18/NW/0545) and informed consent at Addenbrooke’s Hospital, Cambridge. Samples were obtained from two male and three female donors aged 57–83. Participant compensation was not applicable.

### Generation and administration of small circulating ICs

All compounds were administered i.v. via tail vein injection using a 30-G insulin syringe (Becton Dickinson). ICs were prepared in vitro by incubating AF647–OVA (2 mg ml^–1^ in PBS; Invitrogen, O34784) with rabbit polyclonal anti-OVA antiserum (3.7 mg ml^–1^; Sigma-Aldrich, C-6534) at a 1:1 molar ratio (40 μg of OVA + 150 μg of RaOVA) for 60 min in a 37 °C water bath^26^.

### Infectious models

Mice were infected orally with approximately 5 × 10^6^ colony-forming units of *S. enterica* serovar Typhimurium (stain BRD509). Twenty-four hours before infection, mice were gavaged with 20 mg of streptomycin (Bio Basic, SB0494) in 100 μl of PBS to aid colonization. Colony-forming unit counts were verified by streaking the inoculums on to agar plates postgavage. Mice were culled 21 days later by rising CO_2_ concentration.

Urinary tract infection was induced in experimental mice as previously described^52^. Briefly, under isoflurane anesthesia (Baxter), the perineum was cleaned with ethanol, and the urethra was catheterized using 0.28 × 0.60 mm polyethylene tubing (Instech Laboratories) lubricated in sterile Instillagel (CliniMed). In total, 4 × 10^7^ colony-forming units in 100 μl of prepared uropathogenic *E. coli* (strain UTI89) were inoculated into the bladder during one session. Repeated inoculation was performed with 2-h intervals to increase the likelihood of ascending pyelonephritis. Mice were culled 21 days later by rising CO_2_ concentration.

### Isolation of synovium leukocytes from tissues

After death by anesthesia, the right auricles of the mice were cut, and 10 ml of prewarmed 1× PBS was injected into the left ventricle for perfusion. Perfusion was omitted in experiments designed to assess blood samples.

After removal of the skin, the quadriceps femoris muscles were carefully removed. Attachment of the synovium to the bare area of femur was observed by pinching and lifting up the patella with tweezers under a stereoscopic microscope (Stemi 2000-CS, Zeiss). The bone–synovium and meniscus–synovium interface was carefully dissected throughout the knee joint without damaging the bone, and the patella was removed at the end. For flow cytometry analysis, whole-mount synovial tissues were digested with 2 mg ml^–1^ type I collagenase in RPMI and incubated at 37 °C for 45 min. Disaggregated tissue elements were passed through a 70-µm cell strainer.

### Flow cytometry

Measurements were performed on an CytoFLEX LX (Beckman Coulter) and analyzed with FlowJo software (Tree Star). Sorting was performed on an FACSAria Fusion (Becton Dickinson). Single-cell suspensions were incubated with Zombie Aqua (Biolegend) or Viakrome 808 fixable viability dye (Beckman Coulter) diluted 1:250 in PBS for 15 min at 4 °C. Samples were centrifuged and resuspended in FACS buffer supplemented with anti-CD16/CD32 (Biolegend) diluted 1:50, followed by staining with antibodies for 15 min at 4 °C. Antibodies used in this study are listed in Supplementary Table 1.

### Confocal and multiphoton microscopy of mouse samples

Dissected knee joints were fixed in fixative solution made with 9% Glyoxal (Sigma-Aldrich, 128465) and 40% Antigenfix (Diapath, P0016) overnight at 4 °C. For sagittal section imaging, samples were decalcified in 14% EDTA solution for 12 days, followed by 4 h in 30% sucrose in PBS. EDTA was replaced every 72 h. Then, 30-μm sections were blocked and permeabilized in 0.1 M Tris containing 0.3% Triton X-100 (MilliporeSigma), 1% normal mouse serum, 1% normal donkey serum and 1% bovine serum albumin (BSA; R&D Systems). Samples were stained for 2 h at room temperature (RT) in a wet chamber with the appropriate antibodies, washed three times in PBS and mounted in Fluoromount-G (Southern Biotech). For whole-mount imaging, the knee synovium was dissected, as described above, and blocked and permeabilized in 0.1 M Tris containing 0.1% Triton X-100, 1% normal mouse serum, 1% normal donkey serum and 1% BSA in 0.5-ml Eppendorf safe-lock tubes. Samples were stained for 12 h at 4 °C with the appropriate antibodies in 0.5-ml Eppendorf safe-lock tubes, washed three times extensively in PBS and mounted in Fluoromount-G. Images were acquired using a TCS SP8 inverted confocal microscope with a scan format of 512 × 512 (Leica Microsystems) or TCS SP8 3X gated STED confocal inverted microscope (Leica Microsystems) with a ×40/1.3-NA oil or ×40/1.1-NA water objective. Second harmonic generation images were acquired using a Zeiss 710 NLO upright multiphoton microscope equipped with a ×20 water objective and infrared lasers (880 nm) driven by a Ti sapphire laser. Raw imaging data were processed, and a Gaussian filter was applied using Imaris (Bitplane). The antibodies used in this study are listed in Supplementary Table 1.

### Confocal microscopy of human samples

Human synovial specimens were fixed in a fixative solution made with 9% Glyoxal (Sigma-Aldrich, 128465) and 40% Antigenfix (Diapath, P0016) at 4 °C. For sagittal section imaging, samples were placed in 30% sucrose in PBS for 4 h and embedded in OCT embedding matrix (CellPath). Sections were incubated with primary antibodies for 2 h at RT and washed three times in PBS. Sections were then incubated with the appropriate fluorochrome-labeled secondary antibodies for 1 h at RT, washed in PBS and mounted in Fluoromount-G. For whole-mount imaging, synovium samples were dissected into 1 × 1 cm pieces and blocked and permeabilized in 0.1 M Tris containing 0.3% Triton X-100, 1% normal donkey serum, 1% BSA and 0.1% saponin (Sigma). Samples were incubated with primary antibodies for 12 h at 4 °C and washed three times extensively in PBS. Samples were then incubated with the appropriate fluorochrome-labeled secondary antibodies for 12 h at 4 °C and washed three times in PBS. Samples were then embedded in Ce3D solution^30^ for 12 h to clear the tissue and mounted in Ce3D solution. Images were acquired using a TCS SP8 inverted confocal microscope or TCS SP8 3X gated STED confocal inverted microscope on a ×40/1.3-NA oil or ×40/1.1-NA water objective. Raw imaging data were processed, and a Gaussian filter was applied using Imaris (Bitplane). Antibodies used in this study are listed in Supplementary Table 1.

### Iterative staining

Iterative staining of sections was performed as previously described^25,53^. Following acquisition of initial images, the coverslip was removed, and slides were washed three times in PBS to remove mounting medium. Bleaching of the fluorochromes was achieved using a 1 mg ml^–1^ solution of lithium borohydride in water (Acros Organics) for 15 min at RT. The slides or whole-mount synovium were washed three times in PBS before staining with a different set of antibodies. The process was repeated up to three times. Raw imaging data were processed using Imaris (Bitplane), and Hoechst or CD31 was used as fiducial for the alignment of subsequent images.

### Dissection and culture of mouse DRG neurons

DRG neurons were bilaterally excised under a dissection microscope as previously described^54^. For section images, lumbar (L4) or thoracic (T13) DRGs were embedded in OCT embedding matrix, and sections were subsequently made. For culture systems, DRGs were digested with the combined 1.25 mg ml^–1^ collagenase A + 2.5 mg ml^–1^ Dispase ll solution for 30 min at 37 °C. Sensory neurons were cultured on laminin-coated 96-well plates in Neurobasal medium (Thermo Fisher Scientific) supplemented with 1% GlutaMAX (Thermo Fisher Scientific), 50 ng ml^–1^ NGF 2.5S (Gibco) and B-27 supplement (Gibco) for 36 h to remove glial and axon debris that float in the medium. For CGRP release assays, ICs were prepared in vitro by incubating OVA with rabbit polyclonal anti-OVA (1 μg of OVA + 18.5 μg of RaOVA) for 30 min in a 37 °C water bath, spinning down the pellet and washing with PBS three times. Synovium explants were stimulated with OVA or ICs in 200 μl of RPMI for 6 h, and DRG neurons were incubated with the supernatants for 2 h at 37 °C and 5% CO_2_. After incubation, the DRG supernatant was collected and used to quantify the concentration of CGRP using an ELISA kit (Antibodies.com) according to the manufacturer’s instructions.

### CGRP stimulation assay from synovium explants

Whole-mount synovium was dissected from WT mice and incubated with CGRP (100 nM) or vehicle for 4 h. After incubation, the synovium was digested as described above, and macrophages were sorted into 350 μl of Buffer RLT Plus (Qiagen) using a FACSAria Fusion (Becton Dickinson).

### In vivo BIBN4096 treatment

The impact of CGRP signaling on the outcome of systemic IC challenge was evaluated by treating mice with the RAMP1 antagonist BIBN4096 (Tocris), as described previously^16^. Mice were treated by i.p. injection of BIBN4096 (0.5 mg per kg (body weight)) or vehicle 2 h before i.v. IC injection for 2 d in a row, and knee synovium was dissected 48 h after the last injection.

### Cytometric bead array

Concentrations of IL-1β were determined by BD Cytometric Bead Array using the Mouse IL-1β Enhanced Sensitivity Flex Set (562278) according to the manufacturer’s instructions. Bilateral synovial whole mounts were minced in 500 μl of PBS per animal, and samples were acquired using a BD FACSVerse flow cytometer. The results were analyzed by FCAP Array v3 software (BD).

### Bulk RNA-seq library construction and sequencing

The indicated cell types in each experiment were lysed using RLT Plus buffer (Qiagen), vortexed, snap-frozen on dry ice and stored at −80 °C. To extract RNA from cell lysates, an RNeasy Plus Micro kit (Qiagen) was used according to the manufacturer’s instructions. Genomic DNA contamination was removed using Optimal DNA depletion columns (Qiagen). Purified RNA was eluted in nuclease-free water (Ambion) and stored at −80 °C. To assess the quality and concentration of purified RNA, an RNA Pico chip (Applied Biosystems) on a Bioanalyzer 2000 (Applied Biosystems) was used according to the manufacturer’s instructions. For library preparation, a SMARTer Stranded Total RNA-seq Mammalian Pico Input kit v3 (Takara) was used according to the manufacturer’s instructions. Library size was assessed using a High Sensitivity DNA chip (Applied Biosystems) on a Bioanalyzer 2000 (Applied Biosystems) according to the manufacturer’s instructions. Bulk RNA-seq was performed using a Novaseq 6000 (Illumina) on a 2 × 150 bp sequencing run. Pooled libraries were demultiplexed using Casava (Illumina). Fastq files from sequencing libraries were trimmed of the first three nucleotides on the R1 strand. Contaminating adaptor sequences and poor-quality bases were removed using Trim Galore (Babraham Bioinformatics). Libraries were only trimmed for quality. The quality of the resulting files was assessed by FastQC and aligned to the mm10 genome using HISAT2.

Subsequent RNA-seq analysis was performed in the R statistical environment, as described before^55^, with RStudio 2022.02.2. Resulting data are available on the Gene Expression Omnibus (GEO) under accession numbers GSE247475, GSE247476, GSE247477 and GSE272541. Reads were counted and assigned to genes using the Featurecount function from the Rsubread package, and differential expression analysis was performed using DESeq2 with an appropriate design matrix according to the default workflow. Batch effects were removed using the sva package. Figures were plotted using the ggplot2 and pheatmap packages and Prism software. Gene ontology enrichment testing was performed using topGO. GSEA (https://www.gsea-msigdb.org/gsea) was conducted using GSEA v4.3.0 according to developer’s instructions with the preranked option and classic setting. KEGG gene sets were downloaded from the Molecular Signatures Database.

### Analysis of public scRNA-seq datasets

For the analysis of the dataset from GSE145286 (ref. ^6^), raw data from WT mice were downloaded from the GEO archive, and count data were imported into R. After quality control, normalization and dimensional reduction, gene expression was visualized by UMAP. For Extended Data Fig. 1a–c, clusters of endothelial cells were extracted with *Pecam1* and *Cdh5* expression and reclustered. Resulting clusters were annotated using canonical marker gene expression (*Pecam1*, *Sema3g*, *Hey1*, *Podxl*, *Ackr1*, *Vwf* and *Rgcc*), and capillary endothelial cells were further extracted for Fig. 1a–c. Figures were plotted using the EnhancedVolcano and DotPlot functions. For Fig. 7a, cells were clustered using canonical marker genes (*Cd68*, *Lyve1*, *H2-Ab1*, *Cd55*, *Prg4*, *Thy1* and *Pecam1*).

### Development and validation of anti-NP2

Anti-NP2 was newly raised by guinea pigs immunized with peptides Gly 107–Leu 429 (UniProtKB entry O70340).

For immunocytochemistry, HEK293 tsA201 cells (a gift from R. Horn, Thomas Jefferson University) were cultured in high-glucose DMEM (Sigma-Aldrich) containing 10% fetal bovine serum (HyClone), 50 U ml^–1^ penicillin, 50 mg ml^–1^ streptomycin (Invitrogen) and 2 mM l-glutamine at 10% CO_2_ and 37 °C. Cells were transfected with HA-tagged mouse NP1, NP2 or NPR in pCAGGS using Lipofectamine 2000 (Invitrogen). The following day, transfected cells were fixed with 4% paraformaldehyde (PFA) in PBS for 15 min and washed with PBS three times. After blocking/permeabilization with 3% BSA (Sigma-Aldrich) in PBS containing 0.1% Triton X-100 (Sigma-Aldrich) for 30 min, cells were stained with primary antibodies to the HA tag (mouse, 1:1,000, BAbCo, MMS-101P) and NP2CC2 (guinea pig; final concentration of 1 μg ml^–1^) for 2 h at RT, followed by washing with PBS and incubation with the respective secondary antibodies (Alexa 488 (Invitrogen) and Cy3 (Jackson ImmunoResearch Laboratories) to the respective primary antibody; 1:1,000) for 30 min. After washing with PBS, coverslips were mounted on a glass slide with Fluoromount-G (Thermo Fisher Scientific). Fluorescence was detected using an SD-OSR microscope (Olympus).

For immunohistochemistry, mice were housed on a standard light cycle (0800–2000 h) before placement into constant darkness for 7 days. Mice were transcardially perfused with 4% PFA in 0.1 M sodium phosphate buffer (pH 7.2) for 10 min under deep pentobarbital anesthesia in the dark (0 h = control condition) or light-exposed for 4 h (4 h = light-stimulated condition) before death, followed by postfixation of dissected brain samples with 4% PFA in PBS for 2 h at 4 °C. Free-floating sagittal sections (50-μm thickness) from brains fixed in 4% PFA were prepared with a microslicer (DTK-1000, Dosaka EM). Following washing of the sections with PBS containing 0.1% Triton X-100 (wash buffer), sections were subsequently treated with 10% donkey serum for 30 min at RT, incubated with a mixture of primary antibodies (1 μg ml^–1^) diluted in wash buffer overnight, washed three times, incubated with species-specific secondary antibody (Alexa 488, Invitrogen; 1:200) in wash buffer and washed again three times. Finally, sections were attached to glass slides and mounted with VECTASHIELD (Vector Laboratories). Fluorescence images of NP2 were obtained in the visual cortex using a confocal microscope (SD-OSR, Olympus) and a ×10 objective.

### Image analysis

Image analysis was performed using Imaris 9.9.1, ImageJ2 version 2.14.0/1.54f or QuPath as detailed below.

For quantification of the density of each macrophage in the indicated compartments in the synovium, whole-mount synovium images for each macrophage subset were opened in QuPath, and the positions (*x* and *y* axes) of each macrophage were quantified using the ‘Cell detection’ function. The *x* and *y* axes of the center of the synovium were subtracted from the position of each macrophage, and the distance from center was calculated. Macrophage densities were calculated by dividing the number of cells by the indicated area. Density maps were created following the step in the ‘Cell detection’ function using Qupath.

For area quantification, the Imaris analysis tool ‘percentage of material above threshold colocalization’ was used to detect the area of dextran 70 kDa, dextran 2,000 kDa, PV1 over CD31, fluorescently labeled microbeads over PV1 or CD31, CD32b and CGRP. For comparison of different layers of the synovium from whole-mount images, the lining layer was defined by the presence of ERTR7^+^ lining fibroblasts or lining macrophages.

For quantification of penetration depth of synovial sensory fibers, the outermost layer of the synovium (lining layer) was determined by ERTR7 staining, which identifies lining fibroblasts in the synovium. *z*-Stack images of synovial whole mounts were created, and the distances between each fiber (CGRP^+^ and TH^+^ fibers) and the ERTR7^+^ outermost layer were measured and averaged per mouse.

For quantification of the number of clusters of MHCll^+^ macrophages in the whole-mount synovium images, clusters were defined as MHCll^+^ macrophages attached together to form a granulomatous structure with a diameter of >30 μm.

For analysis of the proximity of each macrophage subset to PV1^+^ capillaries, PV1^–^ capillaries and CGRP^+^ nociceptor neurons, we used the Surface module of Imaris (Bitplane)^56^. Briefly, we performed multiparameter imaging of whole-mount synovium using confocal microscopy and reconstructed fluorescent staining of MHCll, CD11c, LYVE1, PV1, CD31 and CGRP separately as three-dimensional volumes using the Surface module and smooth parameters of 1.99 μm for vessels, 1.00 μm for cells and 0.50 μm for neurons. Under the function ‘Set voxel intensity inside surface to 0’, we used PV1 signals as a mask to determine CD31 staining outside of the PV1 volume (defined as ‘CD31^+^PV1^−^ volume’) and CD11c signals as a mask to determine MHCll staining outside of the CD11c volume (defined as ‘MHCll^+^CD11c^−^ volume’). Threshold for absolute intensity and filter for number of voxels were selected to cover signals of the region of interest, and surfaces that do not cover the region of interest were removed. We then quantified the shortest distance between the surface of each macrophage and specific capillaries or neurons, percentage of cells in direct contact with each structure and overlapped volume ratio between each macrophage and neurons. The percentage of cells in the indicated interval of distance and the mean value per animal were calculated.

### Statistical analysis

The results are shown as single data points in a scatter dot plot and as mean ± s.e.m. Between-group differences were determined using a two-tailed *t-*test. A one-way ANOVA with Tukey’s post hoc test was used for comparisons among three or more groups, and a one-way ANOVA with Dunnett’s test was used for comparisons of the mean of each column to the mean of a control column. Statistical analyses were performed using GraphPad Prism 8.4.1 (GraphPad Software). Animal and human sample sizes are indicated on scatter dot plots unless mentioned in the figure legend.

### Reporting summary

Further information on research design is available in the Nature Portfolio Reporting Summary linked to this article.

## Online content

Any methods, additional references, Nature Portfolio reporting summaries, source data, extended data, supplementary information, acknowledgements, peer review information; details of author contributions and competing interests; and statements of data and code availability are available at 10.1038/s41590-024-02011-8.

## Supplementary information

Supplementary Information Supplementary Table 1.

Reporting Summary

## Source data

Source Data Fig. 1 Statistical source data.

Source Data Fig. 2 Statistical source data.

Source Data Fig. 3 Statistical source data.

Source Data Fig. 4 Statistical source data.

Source Data Fig. 5 Statistical source data.

Source Data Fig. 6 Statistical source data.

Source Data Fig. 7 Statistical source data.

Source Data Extended Data Fig. 5 Statistical source data.

Source Data Extended Data Fig. 7 Statistical source data.

Source Data Extended Data Fig. 10 Statistical source data.

## Data Availability

The datasets analyzed during the current study are available from the corresponding author upon reasonable request. Access to raw RNA-seq data related to this study is available through GEO (accession numbers GSE247475, GSE247476, GSE247477 and GSE272541). For the reanalysis of mouse synovium scRNA-seq, we obtained the dataset from GEO (accession number GSE145286). The KEGG gene set is available from Molecular Signatures Database (https://www.gsea-msigdb.org/gsea/msigdb/index.jsp). Source data are provided with this paper.

## Code availability

RNA-seq data analysis was performed in R using field-standard, previously reported packages and indicated parameters, which are described in the Methods and with the relevant source publications cited where appropriate. No custom code beyond adaptation of existing software packages was used in this study. The code is available on reasonable request from the corresponding authors.
