# Supplementary material for: Macrophages and nociceptor neurons form a sentinel unit around fenestrated capillaries to defend the synovium from circulating immune challenge
Source: Nat Immunol. 2024 Nov 25;25(12):2270–83. doi: 10.1038/s41590-024-02011-8 (PMC11588661; doi:10.1038/s41590-024-02011-8)
Supplement: Supplementary file 1 — Supplementary Table 1. [file 41590_2024_2011_MOESM1_ESM.pdf]

nature immunology

Supplementary information https://doi.org/10.1038/s41590-024-02011-8

Macrophages and nociceptor neurons form a sentinel unit around fenestrated capillaries to defend the synovium from circulating immune challenge

In the format provided by the authors and unedited

Table S1

Reagent or resource	Source	Identifier/ dilution
**Antibodies**		
Rat anti-CD16/32	Biolegend	Cat# 156604, Lot# B293349, Clone S17011E, 1/50
Pacific Blue-conjugated rat anti-I-A/I-E	Biolegend	Cat# 107620, Lot#B252427, Clone M5/114.15.2, 1/100
eFluor450-conjugated rat anti-CD3e	Invitrogen	Cat#48-0031-82, Lot#2102873, Clone 145-2C11, 1/100
FITC-conjugated rat anti-CD11b	Biolegend	Cat# 101206, Lot#B286843, Clone M1/70, 1/100
AF488-conjugated rat anti-panendothelial cell antigen	Biolegend	Cat# 120506, Lot#B277044, Clone MECA-32, 1/100
AF488-conjugated mouse anti-tubulin β3	Biolegend	Cat#801203, Lot#B332149, Clone TUJ1, 1/100
AF488-conjugated rat anti-CD68	Biolegend	Cat# 137012, Lot# B272230, Clone FA-11, 1/50
AF488-conjugated rat anti-CD31	Biolegend	Cat# 102514, Lot# B282351, Clone MEC13.3, 1/100
AF488-conjugated rat anti-I-A/I-E	Biolegend	Cat# 107616, Lot# B343353, Clone M5/114.15.2, 1/100
PerCP-Cyanine5.5-conjugated rat anti-Ly6C	Invitrogen	Cat# 45-5932-82, Lot#2162018, Clone HK1.4, 1/200
PerCP-Cyanine5.5-conjugated rat anti-CD206	Biolegend	Cat# 141716, Lot# B270129, Clone C068C2, 1/100
PE-conjugated rat anti-Tim-4	Biolegend	Cat# 130005, Lot# B283682, Clone RMT4-54, 1/100
PE-conjugated Armenian hamster anti-CD11c	Biolegend	Cat# 117308, Lot# B202498, Clone N418, 1/100
PE-conjugated Syrian hamster anti-podoplanin	Biolegend	Cat# 127407, Lot# B328276, Clone 8.1.1, 1/100

PE-conjugated mouse anti-CD32b	Invitrogen	Cat# 12-0321-82, Lot# 2157123, Clone AT130-2, 1/100
PE-conjugated Armenian hamster anti-CD16.2	Biolegend	Cat# 149503, Lot# B273077, Clone 9E9, 1/100
PE-conjugated rat anti-Lyve1	R&D systems	Cat# FAB2125P, Lot# ACFE0220031, Polyclonal, 1/100
PE-Cyanine7-conjugated rat anti-Lyve1	Invitrogen	Cat# 25-0443-80, Lot# 2343412, Clone ALY7, 1/200
PE-Cyanine7-conjugated rat anti-F4/80	Invitrogen	Cat# 25-4801-82, Lot# 2279168, Clone BM8, 1/100
PE-Cyanine7-conjugated rat anti-CD206 (MMR)	Invitrogen	Cat# 25-2061-82, Lot# 2062662, Clone MR6F3, 1/100
eFluor660-conjugated rat anti-Lyve1	Invitrogen	Cat# 50-0443-82, Lot# 2205461, Clone ALY7, 1/100
APC-conjugated rat anti-Ly6C	Invitrogen	Cat# 17-5932-82, Lot# 2002701, Clone HK1.4, 1/200
APC-conjugated rat anti-CD16	Biolegend	Cat# 158005, Lot# B311445, Clone S17014E, 1/100
APC-conjugated mouse anti-CD64	Biolegend	Cat# 139306, Lot# B277148, Clone X54-5/7.1, 1/100
APC-conjugated mouse anti-CD32b	Invitrogen	Cat# 17-0321-80, Lot# 2036645, Clone AT130-2, 1/100
APC-eFluor780-conjugated rat anti-Gr-1	Invitrogen	Cat# 47-5931-82, Lot# 2320762, Clone RB6-8C5, 1/100
AF594-conjugated rat anti-CD31	Biolegend	Cat# 102520, Lot# B368931, Clone MEC13.3, 1/100
AF647-conjugated rat anti-CD31	Biolegend	Cat# 102516, Lot# B308659, Clone MEC13.3, 1/100
AF647-conjugated Armenian hamster anti-CD11c	Biolegend	Cat# 117312, Lot# B341497, Clone N418, 1/100
AF647-conjugated rat anti-B220	BD Pharmingen	Cat# 557683, Lot# 9123764, Clone RA3-6B2, 1/100

AF647-conjugated rat anti-ER-TR7	Novus Biologicals	Cat# NB100-64932AF647, Lot# D102142, Clone ER-TR7, 1/100
APC-Cy7-conjugated rat anti-Ly6G	BD Pharmingen	Cat# 560600, Lot# 8277987, Clone 1A8, 1/100
Brilliant Violet421-conjugated rat anti-F4/80	Biolegend	Cat# 123131, Lot# B258771, Clone BM8, 1/100
Brilliant Violet605-conjugated rat anti-I-A/I-E	Biolegend	Cat# 107639, Lot# B293222, Clone M5/114.15.2, 1/100
Brilliant Violet650-conjugated mouse anti-CX3CR1	Biolegend	Cat# 149033, Lot# B301229, Clone SA011F11, 1/100
Goat anti-CGRP	Abcam	Cat# ab36001, Lot# GR3445403-5, Polyclonal, 1/200
Rabbit anti-tyrosine hydroxylase	Abcam	Cat# ab112, Lot# GR3435522-1, Polyclonal, 1/200
Rabbit anti-tubβ3	Abcam	Cat# ab18207, Lot# GR3257458-1, polyclonal, 1/200
Biotin-conjugated mouse anti- Ea52-68 peptide bound to I-Ab	Invitrogen	Cat# 13-5741-82, Lot# 1947272, Clone YAe, 1/100
Guinea pig anti-NP2	In house	NA
AF488-conjugated mouse anti-alpha smooth muscle actin	Abcam	Cat# AB184675, Lot# 1040301-1, Clone 1A4, 1/100
AF594-conjugated donkey anti-guinea Pig	Jackson ImmunoResearch	Cat# 706-585-148, Polyclonal, 1/200
AF594-conjugated mouse anti-CD31	Biolegend	Cat# 303126, Lot# B297139, Clone WN59, 1/100
AF647-conjugated mouse anti-CD55	Novus Biologicals	Cat# NBP2-47964AF647, Lot# D105865, Clone 143-30, 1/100
AF647-conjugated mouse anti-HLA-DR	Abcam	Cat# ab223907, Lot# GR3441855-1, Clone TAL1B5, 1/100
Goat anti-CD32B	Abcam	Cat# AB77093, Lot# 1034248-3, Polyclonal, 1/100
Goat anti-LYVE1	R&D systems	Cat# AF2089, Lot# KPY0119121, Polyclonal, 1/100

Rabbit anti-PLVAP	Novus Biologicals	Cat# NBP1-83911, Lot# 000007304, Polyclonal, 1/100
Rabbit anti-LYVE1	Abcam	Cat# ab33682, Lot# GR295168-4, polyclonal, 1/100
PE/Dazzle594-conjugated mouse anti-CD206	Biolegend	Cat# 321130, Lot# B271255, Clone 15-2, 1/100
Anti-mouse CSF1R (CD115)	Biocell	Cat# BE0213, Lot# 808022M2, Clone AFS98
Anti-mouse TNFα	Biocell	Cat# BE0058, Lot# 728222J1, Clone XT3.11
Anti-mouse CXCL1	R&D systems	Cat# MAB453, Lot#AOS0823041, Clone 48415
Anti-mouse IL-1β	Invivogen	Cat# mil1b-mab9-02, Lot# 10594-44-01
Rat IgG2a isotype control	Biocell	Cat#BE0089, Lot# 849322J2, Clone 2A3
**Chemicals, peptides, and recombinant proteins**		
Glyoxal	Sigma-Aldrich	Cat# 128465
Antigenfix	Diapath	Cat# P0016
Reagent diluent concentrate 2	R&D systems	Cat# DY995
Saponin	Sigma	Cat# SAE0073
Neurobasal medium	Thermo Scientific	Cat# 10888022
Glutamax Supplement	Gibco	Cat# 35050061
B-27 supplement (50x)	Gibco	Cat# 17504044
NGF 2.5S	Gibco	Cat# 13257-019
Collagenase, type1, powder	Thermo Scientific	Cat# 17100017
Eα (52–68) peptide	Anaspec	Cat# AS-61621
Dextran, Texas Red™, 70,000 MW	Invitrogen	Cat# D1864
Dextran, Fluorescein, 2,000,000 MW	Invitrogen	Cat# D7137
Fluospheres™ Carboxylate-Modified Microspheres, 0.2 µm, orange fluorescent (540/560)	Thermo Scientific	Cat# F8809
Fluospheres™ Carboxylate-Modified Microspheres, 2.0 µm, yellow-green fluorescent (505/515)	Thermo Scientific	Cat# F8827
Fluospheres™ Carboxylate-Modified Microspheres, 0.04 µm, dark red fluorescent (660/680)	Thermo Scientific	Cat# F8789
CGRP (mouse, rat)	Cambridge Research Biochemicals	Cat# crb1000889h
BIBN 4096	Tocris	Cat# 4561
Diclofenac sodium salt	Sigma-Aldrich	Cat# D6899
A438079 hydrochloride	Tocris	Cat# 2972
**Commercial assays**		

Mouse CGRP ELISA kit	Antibodies.com	Cat# A76318
Ce3D Tissue Clearing kit	Biolegend	Cat# 427701
SMARTer® Stranded Total RNA-Seq Kit v3 - Pico Input Mammalian	Takara Bio	Cat#634485
Mouse CXCL1/KC DuoSet ELISA	R&D systems	Cat# DY453-05
Mouse CXCL2/MIP-2 DuoSet ELISA	R&D systems	Cat# DY452-05
**Software and algorithms**		
Imaris	Version 9.9.1	
FlowJo	Version 10.6.2	
ImageJ2	Version 2.14.0/1.54f	
QuPath	Version 0.3.2	
