# Supplementary material for: Macrophages and nociceptor neurons form a sentinel unit around fenestrated capillaries to defend the synovium from circulating immune challenge
Source: Nat Immunol. 2024 Nov 25;25(12):2270–83. doi: 10.1038/s41590-024-02011-8 (PMC11588661; doi:10.1038/s41590-024-02011-8)
Supplement: Supplementary file 2 — Reporting Summary [file 41590_2024_2011_MOESM2_ESM.pdf]

nature portfolio

Corresponding author(s): Tetsuo Hasegawa

Last updated by author(s): 30/9/2024

Reporting Summary

Nature Portfolio wishes to improve the reproducibility of the work that we publish. This form provides structure for consistency and transparency in reporting. For further information on Nature Portfolio policies, see our Editorial Policies and the Editorial Policy Checklist.

Statistics

For all statistical analyses, confirm that the following items are present in the figure legend, table legend, main text, or Methods section.

n/a	Confirmed	
☐	☒	The exact sample size (*n*) for each experimental group/condition, given as a discrete number and unit of measurement
☐	☒	A statement on whether measurements were taken from distinct samples or whether the same sample was measured repeatedly
☐	☒	The statistical test(s) used AND whether they are one- or two-sided<br>*Only common tests should be described solely by name; describe more complex techniques in the Methods section.*
☒	☐	A description of all covariates tested
☐	☒	A description of any assumptions or corrections, such as tests of normality and adjustment for multiple comparisons
☐	☒	A full description of the statistical parameters including central tendency (e.g. means) or other basic estimates (e.g. regression coefficient) AND variation (e.g. standard deviation) or associated estimates of uncertainty (e.g. confidence intervals)
☐	☒	For null hypothesis testing, the test statistic (e.g. *F*, *t*, *r*) with confidence intervals, effect sizes, degrees of freedom and *P* value noted<br>*Give P values as exact values whenever suitable.*
☒	☐	For Bayesian analysis, information on the choice of priors and Markov chain Monte Carlo settings
☒	☐	For hierarchical and complex designs, identification of the appropriate level for tests and full reporting of outcomes
☒	☐	Estimates of effect sizes (e.g. Cohen's *d*, Pearson's *r*), indicating how they were calculated

Our web collection on statistics for biologists contains articles on many of the points above.

Software and code

Policy information about availability of computer code

Data collection	CytoFLEX LX (Beckman Coulter) for FACS, FACSAria Fusion (Becton Dickinson) for cell sorting, TCS SP8 inverted confocal microscope (Leica Microsystems) for confocal imaging, TCS SP8 3X gated STED confocal inverted microscope (Leica Microsystems) for confocal imaging, Zeiss 710 NLO upright multiphoton microscope (Zeiss) for multiphoton imaging, stereoscopic microscope (Stemi 2000-CS, Zeiss) for synovium dissection, Illumina NovaSeq 6000 for RNA sequencing. BD FACSVerse flow cytometer (Becton Dickinson) for cytometric bead array.
Data analysis	Imaris version 9.9.1, FlowJo version 10.6.2, ImageJ2 version 2.14.0/1.54f, QuPath version 0.3.2, Prism version 8.4.1, FCAP Array v3 software. RNA-seq analysis was performed in the R statistical environment with RStudio 2022.02.2. Resulting data is available on GEO under accession numbers GSE247475, GSE247476, GSE247477, and GSE272541. Reads were counted and assigned to genes using the Featurecount function from the Rsubread package and differential expression analysis was performed using DESeq2 with an appropriate design matrix according to the default workflow, and batch effects removed using the sva package. Figures were plotted using the ggplot2, pheatmap packages and Prism software. Gene ontology enrichment testing was performed using topGO. Gene Set Enrichment Analysis (GSEA, https://www.gsea-msigdb.org/gsea) was conducted using GSEA v4.3.0 according to developers' instruction, using the pre-ranked option and classic setting. Kegg gene set was downloaded from Molecular Signature Database (MSigDB). No custom code beyond adaptation of existing software packages were used in this study. The code is available on reasonable request from the corresponding author.

For manuscripts utilizing custom algorithms or software that are central to the research but not yet described in published literature, software must be made available to editors and reviewers. We strongly encourage code deposition in a community repository (e.g. GitHub). See the Nature Portfolio guidelines for submitting code & software for further information.

Data

Policy information about availability of data

All manuscripts must include a data availability statement. This statement should provide the following information, where applicable:

- Accession codes, unique identifiers, or web links for publicly available datasets
- A description of any restrictions on data availability
- For clinical datasets or third party data, please ensure that the statement adheres to our policy

Access to raw RNA-seq data related to this study is available through the Gene Expression Omnibus (GEO) (accession number: GSE247475, GSE247476, GSE247477, and GSE272541). For the reanalysis of mouse synovium single-cell RNA sequencing, we obtained the dataset from GEO (the accession number: GSE145286). Kegg gene set is available from Molecular Signature Database (https://www.gsea-msigdb.org/gsea/msigdb/index.jsp). Source Data are provided with this paper.

Research involving human participants, their data, or biological material

Policy information about studies with human participants or human data. See also policy information about sex, gender (identity/presentation), and sexual orientation and race, ethnicity and racism.

Reporting on sex and gender	In human study, samples were collected without regard to gender.
Reporting on race, ethnicity, or other socially relevant groupings	Because of the recruitment protocol stated below, samples from UK citizens were only used in this study.
Population characteristics	Human synovial specimens were obtained from osteoarthritis patients undergoing replacement surgery or synovectomy with prior ethical approval (REC: 18/NW/0545) and informed consent at Addenbrooke's Hospital, Cambridge. Samples were obtained from 2 male and 3 female donors aged 57-83.
Recruitment	Human synovial specimens were obtained from osteoarthritis patients undergoing replacement surgery or synovectomy. The presence of osteoarthritis and their age distriution may have potential bias to the results, which can show more inflammatory phenotye than healthy subjects.
Ethics oversight	Researches using human subjects in this study are approved with prior ethical approval (REC: 18/NW/0545) at Addenbrooke's Hospital, Cambridge.

Note that full information on the approval of the study protocol must also be provided in the manuscript.

Field-specific reporting

Please select the one below that is the best fit for your research. If you are not sure, read the appropriate sections before making your selection.

☒ Life sciences ☐ Behavioural & social sciences ☐ Ecological, evolutionary & environmental sciences

For a reference copy of the document with all sections, see nature.com/documents/nr-reporting-summary-flat.pdf

Life sciences study design

All studies must disclose on these points even when the disclosure is negative.

Sample size	We did not use statistical methods to predetermine sample sizes. We estimated the required sample sizes by considering variations and means of preliminary results, and sought to reach reliable conclusions with as small sample size as possible. Previously published results, experimental complexity, the cost of experiments and past experiences were used to determine the sample sizes although we did not refer to any specific previous study.
Data exclusions	No data were excluded.
Replication	Experiments included sufficient sample size to ensure the reproducibility of the findings. Representative data was confirmed at least twice by performing independent experiments. All attempts at replication were successful.
Randomization	The animals were randomly assigned to each treatment/control group within each genotype. For human experiments, parameters were compared within the specimen from same patients. Therefore, randomisation wasn't applied.
Blinding	Investigators were aware of the group allocation because the treatment groups needed to be clear when performing the experiments.

Reporting for specific materials, systems and methods

We require information from authors about some types of materials, experimental systems and methods used in many studies. Here, indicate whether each material, system or method listed is relevant to your study. If you are not sure if a list item applies to your research, read the appropriate section before selecting a response.

Materials & experimental systems

n/a	Involved in the study
☐	☒ Antibodies
☐	☒ Eukaryotic cell lines
☒	☐ Palaeontology and archaeology
☐	☒ Animals and other organisms
☒	☐ Clinical data
☒	☐ Dual use research of concern
☒	☐ Plants

Methods

n/a	Involved in the study
☒	☐ ChIP-seq
☐	☒ Flow cytometry
☒	☐ MRI-based neuroimaging

Antibodies

Antibodies used

Information of all the antibodies used in this study is provided in detail (catalog number, clone type, lot number, supplier name) in Supplementary Table 1.
Rat anti-CD16/32 Biolegend Cat# 156604, Lot# B293349, Clone S17011E
Pacific Blue-conjugated rat anti-I-A/I-E Biolegend Cat# 107620, Lot#B252427, Clone M5/114.15.2
eFluor450-conjugated rat anti-CD3e Invitrogen Cat#48-0031-82, Lot#2102873, Clone 145-2C11
FITC-conjugated rat anti-CD11b Biolegend Cat# 101206, Lot#B286843, Clone M1/70
AF488-conjugated rat anti-panendothelial cell antigen Biolegend Cat# 120506, Lot#B277044, Clone MECA-32
AF488-conjugated mouse anti-tubulin β3 Biolegend Cat#801203, Lot#B332149, Clone TUJ1
AF488-conjugated rat anti-CD68 Biolegend Cat# 137012, Lot# B272230, Clone FA-11
AF488-conjugated rat anti-CD31 Biolegend Cat# 102514, Lot# B282351, Clone MEC13.3
AF488-conjugated rat anti-I-A/I-E Biolegend Cat# 107616, Lot# B343353, Clone M5/114.15.2
PerCP-Cyanine5.5-conjugated rat anti-Ly6C Invitrogen Cat# 45-5932-82, Lot#2162018, Clone HK1.4
PerCP-Cyanine5.5-conjugated rat anti-CD206 Biolegend Cat# 141716, Lot# B270129, Clone C068C2
PE-conjugated rat anti-Tim-4 Biolegend Cat# 130005, Lot# B283682, Clone RMT4-54
PE-conjugated Armenian hamster anti-CD11c Biolegend Cat# 117308, Lot# B202498, Clone N418
PE-conjugated Syrian hamster anti-podoplanin Biolegend Cat# 127407, Lot# B328276, Clone 8.1.1
PE-conjugated mouse anti-CD32b Invitrogen Cat# 12-0321-82, Lot# 2157123, Clone AT130-2
PE-conjugated Armenian hamster anti-CD16.2 Biolegend Cat# 149503, Lot# B273077, Clone 9E9
PE-conjugated rat anti-Lyve1 R&D systems Cat# FAB2125P, Lot# ACFE0220031, Polyclonal
PE-Cyanine7-conjugated rat anti-Lyve1 Invitrogen Cat# 25-0443-80, Lot# 2343412, Clone ALY7
PE-Cyanine7-conjugated rat anti-F4/80 Invitrogen Cat# 25-4801-82, Lot# 2279168, Clone BM8
PE-Cyanine7-conjugated rat anti-CD206 (MMR) Invitrogen Cat# 25-2061-82, Lot# 2062662, Clone MR6F3
eFluor660-conjugated rat anti-Lyve1 Invitrogen Cat# 50-0443-82, Lot# 2205461, Clone ALY7
APC-conjugated rat anti-Ly6C Invitrogen Cat# 17-5932-82, Lot# 2002701, Clone HK1.4
APC-conjugated rat anti-CD16 Biolegend Cat# 158005, Lot# B311445, Clone S17014E
APC-conjugated mouse anti-CD64 Biolegend Cat# 139306, Lot# B277148, Clone X54-5/7.1
APC-conjugated mouse anti-CD32b Invitrogen Cat# 17-0321-80, Lot# 2036645, Clone AT130-2
APC-eFluor780-conjugated rat anti-Gr-1 Invitrogen Cat# 47-5931-82, Lot# 2320762, Clone RB6-8C5
AF594-conjugated rat anti-CD31 Biolegend Cat# 102520, Lot# B368931, Clone MEC13.3
AF647-conjugated rat anti-CD31 Biolegend Cat# 102516, Lot# B308659, Clone MEC13.3
AF647-conjugated Armenian hamster anti-CD11c Biolegend Cat# 117312, Lot# B341497, Clone N418
AF647-conjugated rat anti-B220 BD Pharmingen Cat# 557683, Lot# 9123764, Clone RA3-6B2
AF647-conjugated rat anti-ER-TR7 Novus Biologicals Cat# NB100-64932AF647, Lot# D102142, Clone ER-TR7
APC-Cy7-conjugated rat anti-Ly6G BD Pharmingen Cat# 560600, Lot# 8277987, Clone 1A8
Brilliant Violet421-conjugated rat anti-F4/80 Biolegend Cat# 123131, Lot# B258771, Clone BM8
Brilliant Violet605-conjugated rat anti-I-A/I-E Biolegend Cat# 107639, Lot# B293222, Clone M5/114.15.2
Brilliant Violet650-conjugated mouse anti-CX3CR1 Biolegend Cat# 149033, Lot# B301229, Clone SA011F11
Goat anti-CGRP Abcam Cat# ab36001, Lot# GR3445403-5, Polyclonal
Rabbit anti-tyrosine hydroxylase Abcam Cat# ab112, Lot# GR3435522-1, Polyclonal
Rabbit anti-tubβ3 Abcam Cat# ab18207, Lot# GR3257458-1, polyclonal
Biotin-conjugated mouse anti- Ea52-68 peptide bound to I-Ab Invitrogen Cat# 13-5741-82, Lot# 1947272, Clone YAe
Guinea pig anti-NP2 In house NA
AF488-conjugated mouse anti-alpha smooth muscle actin Abcam Cat# AB184675, Lot# 1040301-1, Clone 1A4
AF594-conjugated donkey anti-guinea Pig Jackson ImmunoResearch Cat# 706-585-148, Polyclonal
AF594-conjugated mouse anti-CD31 Biolegend Cat# 303126, Lot# B297139, Clone WN59
AF647-conjugated mouse anti-CD55 Novus Biologicals Cat# NBP2-47964AF647, Lot# D105865, Clone 143-30
AF647-conjugated mouse anti-HLA-DR Abcam Cat# ab223907, Lot# GR3441855-1, Clone TAL1B5
Goat anti-CD32B Abcam Cat# AB77093, Lot# 1034248-3, Polyclonal
Goat anti-LYVE1 R&D systems Cat# AF2089, Lot# KPY0119121, Polyclonal
Rabbit anti-PLVAP Novus Biologicals Cat# NBP1-83911, Lot# 000007304, Polyclonal
Rabbit anti-LYVE1 Abcam Cat# ab33682, Lot# GR295168-4, polyclonal
PE/Dazzle594-conjugated mouse anti-CD206 Biolegend Cat# 321130, Lot# B271255, Clone 15-2
Anti-mouse CSF1R (CD115) Biocell Cat# BE0213, Lot# 808022M2, Clone AFS98
Anti-mouse TNFα Biocell Cat# BE0058, Lot# 728222J1, Clone XT3.11
Anti-mouse CXCL1 R&D systems Cat# MAB453, Lot#AOS0823041, Clone 48415

Anti-mouse IL-1β Invivogen Cat# mil1b-mab9-02, Lot# 10594-44-01, Clone 7E3
Dilution of each antibody is provided in supplementary table 1.

Validation

Antibodies used in this study are commercially available and have been validated by the manufacturers. Validation statements are provided on the manufacture's website.

Rat anti-CD16/32 Biolegend Cat# 156604
https://www.biolegend.com/en-gb/products/trustain-fcx-plus-anti-mouse-cd16-32-antibody-17085

Pacific Blue-conjugated rat anti-I-A/I-E Biolegend Cat# 107620
https://www.biolegend.com/en-gb/products/pacific-blue-anti-mouse-i-a-i-e-antibody-3136?GroupID=BLG11931

eFluor450-conjugated rat anti-CD3e Invitrogen Cat#48-0031-82
https://www.thermofisher.com/antibody/product/CD3e-Antibody-clone-145-2C11-Monoclonal/48-0031-82

FITC-conjugated rat anti-CD11b Biolegend Cat# 101206
https://www.biolegend.com/en-gb/search-results/fitc-anti-mouse-human-cd11b-antibody-347?GroupID=BLG10660

AF488-conjugated rat anti-panendothelial cell antigen Biolegend Cat# 120506
https://www.biolegend.com/en-gb/products/alexa-fluor-488-anti-mouse-panendothelial-cell-antigen-antibody-3074

AF488-conjugated mouse anti-tubulin β3 Biolegend Cat#801203
https://www.biolegend.com/nl-be/products/alexa-fluor-488-anti-tubulin-beta-3-tubb3-antibody-10828?GroupID=GROUP686

AF488-conjugated rat anti-CD68 Biolegend Cat# 137012
https://www.biolegend.com/nl-be/products/alexa-fluor-488-anti-mouse-cd68-antibody-6619

AF488-conjugated rat anti-CD31 Biolegend Cat# 102514
https://www.biolegend.com/nl-be/products/alexa-fluor-488-anti-mouse-cd31-antibody-3093

AF488-conjugated rat anti-I-A/I-E Biolegend Cat# 107616
https://www.biolegend.com/nl-be/products/alexa-fluor-488-anti-mouse-i-a-i-e-antibody-3134

PerCP-Cyanine5.5-conjugated rat anti-Ly6C Biolegend Cat# 45-5932-82
https://www.thermofisher.com/antibody/product/Ly-6C-Antibody-clone-HK1-4-Monoclonal/45-5932-82

PerCP-Cyanine5.5-conjugated rat anti-CD206 Biolegend Cat# 141716
https://www.biolegend.com/en-gb/products/percp-cyanine5-5-anti-mouse-cd206-mmr-antibody-8477?GroupID=BLG9506

PE-conjugated rat anti-Tim-4 Biolegend Cat# 130005
https://www.biolegend.com/en-gb/products/pe-anti-mouse-tim-4-antibody-5242

PE-conjugated Armenian hamster anti-CD11c Biolegend Cat# 117308
https://www.biolegend.com/en-gb/products/pe-anti-mouse-cd11c-antibody-1816

PE-conjugated Syrian hamster anti-podoplanin Biolegend Cat# 127407
https://www.biolegend.com/en-gb/products/pe-anti-mouse-podoplanin-antibody-4882

PE-conjugated mouse anti-CD32b Invitrogen Cat# 12-0321-82
https://www.thermofisher.com/antibody/product/CD32b-Antibody-clone-AT130-2-Monoclonal/12-0321-82

PE-conjugated Armenian hamster anti-CD16.2 Biolegend Cat# 149503
https://www.biolegend.com/en-gb/products/pe-anti-mouse-cd16-2-fcgammariv-antibody-11913?GroupID=BLG13687

PE-conjugated rat anti-Lyve1 R&D systems Cat# FAB2125P
https://www.rndsystems.com/products/mouse-lyve-1-pe-conjugated-antibody-223322_fab2125p

PE-Cyanine7-conjugated rat anti-Lyve1 Invitrogen Cat# 25-0443-80
https://www.thermofisher.com/antibody/product/LYVE1-Antibody-clone-ALY7-Monoclonal/25-0443-82

PE-Cyanine7-conjugated rat anti-F4/80 Invitrogen Cat# 25-4801-82
https://www.thermofisher.com/antibody/product/F4-80-Antibody-clone-BM8-Monoclonal/25-4801-82

PE-Cyanine7-conjugated rat anti-CD206 (MMR) Invitrogen Cat# 25-2061-82
https://www.thermofisher.com/antibody/product/CD206-MMR-Antibody-clone-MR6F3-Monoclonal/25-2061-82

eFluor660-conjugated rat anti-Lyve1 Invitrogen Cat# 50-0443-82
https://www.thermofisher.com/antibody/product/LYVE1-Antibody-clone-ALY7-Monoclonal/50-0443-82

APC-conjugated rat anti-Ly6C Invitrogen Cat# 17-5932-82
https://www.thermofisher.com/antibody/product/Ly-6C-Antibody-clone-HK1-4-Monoclonal/17-5932-82

APC-conjugated rat anti-CD16 Biolegend Cat# 158005
https://www.biolegend.com/en-gb/products/apc-anti-mouse-cd16-antibody-19298

APC-conjugated mouse anti-CD64 Biolegend Cat# 139306

https://www.biolegend.com/en-gb/products/apc-anti-mouse-cd64-fcgammari-antibody-7874?GroupID=BLG8810

APC-conjugated mouse anti-CD32b Invitrogen Cat# 17-0321-80
https://www.thermofisher.com/antibody/product/CD32b-Antibody-clone-AT130-2-Monoclonal/17-0321-82

APC-eFluor780-conjugated rat anti-Gr-1 Invitrogen Cat# 47-5931-82
https://www.thermofisher.com/antibody/product/Ly-6G-Ly-6C-Antibody-clone-RB6-8C5-Monoclonal/47-5931-82

AF594-conjugated rat anti-CD31 Biolegend Cat# 102520
https://www.biolegend.com/en-gb/products/alexa-fluor-594-anti-mouse-cd31-antibody-9633?GroupID=BLG10559

AF647-conjugated rat anti-CD31 Biolegend Cat# 102516
https://www.biolegend.com/en-gb/products/alexa-fluor-647-anti-mouse-cd31-antibody-3094

AF647-conjugated Armenian hamster anti-CD11c Biolegend Cat# 117312
https://www.biolegend.com/en-gb/products/alexa-fluor-647-anti-mouse-cd11c-antibody-2703

AF647-conjugated rat anti-B220 BD Pharmingen Cat# 557683
https://www.bdbiosciences.com/en-us/products/reagents/flow-cytometry-reagents/research-reagents/single-color-antibodies-ruo/alexa-fluor-647-rat-anti-mouse-cd45r.557683

AF647-conjugated rat anti-ER-TR7 Novus Biologicals Cat# NB100-64932AF647
https://www.novusbio.com/products/fibroblast-antibody-er-tr7_nb100-64932af647

APC-Cy7-conjugated rat anti-Ly6G BD Pharmingen Cat# 560600
https://www.bdbiosciences.com/en-us/products/reagents/flow-cytometry-reagents/research-reagents/single-color-antibodies-ruo/apc-cy-7-rat-anti-mouse-ly-6g.560600

Brilliant Violet421-conjugated rat anti-F4/80 Biolegend Cat# 123131
https://www.biolegend.com/fr-ch/products/brilliant-violet-421-anti-mouse-f4-80-antibody-7199?GroupID=BLG5319

Brilliant Violet605-conjugated rat anti-I-A/I-E Biolegend Cat# 107639
https://www.biolegend.com/fr-ch/products/brilliant-violet-605-anti-mouse-i-a-i-e-antibody-11988

Brilliant Violet650-conjugated mouse anti-CX3CR1 Biolegend Cat# 149033
https://www.biolegend.com/fr-ch/products/brilliant-violet-650-anti-mouse-cx3cr1-antibody-12121

Goat anti-CGRP Abcam Cat# ab36001
https://www.abcam.com/en-gb/products/primary-antibodies/cgrp-antibody-ab36001

Rabbit anti-tyrosine hydroxylase Abcam Cat# ab112
https://www.abcam.com/en-gb/products/primary-antibodies/tyrosine-hydroxylase-antibody-neuronal-marker-ab112

Rabbit anti-tubβ3 Abcam Cat# ab18207,
https://www.abcam.com/en-gb/products/primary-antibodies/beta-iii-tubulin-antibody-neuronal-marker-ab18207

Biotin-conjugated mouse anti- Ea52-68 peptide bound to I-Ab Invitrogen Cat# 13-5741-82
https://www.thermofisher.com/antibody/product/Ea52-68-peptide-bound-to-I-Ab-Antibody-clone-eBioY-Ae-YAe-Y-Ae-Monoclonal/13-5741-82

AF488-conjugated mouse anti-alpha smooth muscle actin Abcam Cat# AB184675
https://www.abcam.com/en-gb/products/primary-antibodies/alexa-fluor-488-alpha-smooth-muscle-actin-antibody-1a4-ab184675

AF594-conjugated donkey anti-guinea Pig Jackson ImmunoResearch Cat# 706-585-148
https://www.jacksonimmuno.com/catalog/products/706-585-148

AF594-conjugated mouse anti-CD31 Biolegend Cat# 303126
https://www.biolegend.com/en-gb/products/alexa-fluor-594-anti-human-cd31-antibody-10182?GroupID=BLG10311

AF647-conjugated mouse anti-CD55 Novus Biologicals Cat# NBP2-47964AF647
https://www.novusbio.com/products/cd55-daf-antibody-143-30_nbp2-47964af647

AF647-conjugated mouse anti-HLA-DR Abcam Cat# ab223907
https://www.abcam.com/en-gb/products/primary-antibodies/alexa-fluor-647-hla-dr-antibody-tal-1b5-ab223907

Goat anti-CD32B Abcam Cat# AB77093
https://www.abcam.com/en-gb/products/primary-antibodies/cd32b-antibody-ab77093

Goat anti-LYVE1 R&D systems Cat# AF2089
https://www.rndsystems.com/products/human-lyve-1-antibody_af2089

Rabbit anti-PLVAP Novus Biologicals Cat# NBP1-83911
https://www.novusbio.com/products/plvap-antibody_nbp1-83911

Rabbit anti-LYVE1 Abcam Cat# ab33682
https://www.abcam.com/en-gb/products/primary-antibodies/lyve1-antibody-ab33682

PE/Dazzle594-conjugated mouse anti-CD206 Biolegend Cat# 321130
https://www.biolegend.com/en-gb/products/pe-dazzle-594-anti-human-cd206-mmr-antibody-13265?GroupID=BLG4585

Anti-mouse CSF1R (CD115) Biocell Cat# BE0213
https://bioxcell.com/invivomab-anti-mouse-csf1r-cd115-be0213

Anti-mouse TNFα Biocell Cat# BE0058
https://bioxcell.com/invivomab-anti-mouse-tnf-alpha-be0058

Anti-mouse CXCL1 R&D systems Cat# MAB453
https://www.rndsystems.com/products/mouse-cxcl1-groalpha-kc-cinc-1-antibody-48415_mab453

Anti-mouse IL-1β Invivogen Cat# mil1b-mab9-02
https://www.invivogen.com/recombinant-anti-mouse-il1beta-antibody

Validation data of Guinea pig anti-NP2 is provided in Supplementary fig. 9.

Eukaryotic cell lines

Policy information about cell lines and Sex and Gender in Research

Cell line source(s)	HEK293 tsA201 cells were kindly gifted from R. Horn, Thomas Jefferson University, Philadelphia, USA.
Authentication	Authentication has originally been performed by the providor.
Mycoplasma contamination	Cell lines were regularly tested for mycoplasma contamination.
Commonly misidentified lines (See ICLAC register)	*Name any commonly misidentified cell lines used in the study and provide a rationale for their use.*

Animals and other research organisms

Policy information about studies involving animals; ARRIVE guidelines recommended for reporting animal research, and Sex and Gender in Research

Laboratory animals	Wild-type mice (C57BL/6J background) were bred in-house or purchased from Jackson Laboratories (Margate, UK). Transgenic mice expressing Venus EYFP under the control of the CD11c promoter were a gift from M Nussenzweig (Rockefeller University, New York, New York, USA). Fcgr2b−/− mice were kindly provided by J. Ravetch (Rockefeller University) and S. Bolland (US National Institutes of Health, US National Institute of Allergy and Infectious Diseases (NIAID)). CX3CR1-cre/ERT2: IL-1βflox mice were provided by Dr. Denes. Both male and female mice were used. For in vivo experiments, 8- to 20-week-old mice were used unless mentioned. Mice were maintained in specific pathogen-free conditions at a Home Office-approved facility with controlled humidity and temperature with a light/dark cycle of 12h each in the UK.
Wild animals	No wild animals were involved.
Reporting on sex	Both female and male mice were used in the study and no sex difference was confirmed.
Field-collected samples	No samples were collected from the field.
Ethics oversight	All procedures were ethically approved by the University of Cambridge Animal Welfare and Ethical Review Body and carried out in accordance with the United Kingdom Animals (Scientific Procedures) Act of 1986 under the authority of a UK Home Office Licence.

Note that full information on the approval of the study protocol must also be provided in the manuscript.

Plants

Seed stocks	*Report on the source of all seed stocks or other plant material used. If applicable, state the seed stock centre and catalogue number. If plant specimens were collected from the field, describe the collection location, date and sampling procedures.*
Novel plant genotypes	*Describe the methods by which all novel plant genotypes were produced. This includes those generated by transgenic approaches, gene editing, chemical/radiation-based mutagenesis and hybridization. For transgenic lines, describe the transformation method, the number of independent lines analyzed and the generation upon which experiments were performed. For gene-edited lines, describe the editor used, the endogenous sequence targeted for editing, the targeting guide RNA sequence (if applicable) and how the editor was applied.*
Authentication	*Describe any authentication procedures for each seed stock used or novel genotype generated. Describe any experiments used to assess the effect of a mutation and, where applicable, how potential secondary effects (e.g. second site T-DNA insertions, mosiacism, off-target gene editing) were examined.*

Flow Cytometry

Plots

Confirm that:

☒ The axis labels state the marker and fluorochrome used (e.g. CD4-FITC).

☒ The axis scales are clearly visible. Include numbers along axes only for bottom left plot of group (a 'group' is an analysis of identical markers).

☒ All plots are contour plots with outliers or pseudocolor plots.

☒ A numerical value for number of cells or percentage (with statistics) is provided.

Methodology

Sample preparation	After sacrifice under anaesthesia, the right auricles of the mice were cut and 10 ml of pre-warmed 1× PBS was injected into the left ventricle for perfusion. Perfusion was omitted in experiments designed to assess blood samples.<br>After removal of the skin, the quadriceps femoris muscles were carefully removed. The attachment of synovium to the bare area of femur was observed by pinching and lifting up the patella with tweezers under a stereoscopic microscope (Stemi 2000-CS, Zeiss). The bone-synovium and the meniscus-synovium interface is carefully dissected throughout knee joint without damaging the bone, and patella is removed at the end. For flow cytometry analysis, whole mount synovial tissues were digested with 2 mg/ml type I collagenase in RPMI and incubated at 37°C for 45 min. Disaggregated tissue elements were passed through a 70 µm cell strainer.<br>Measurements were performed on an CytoFLEX LX (Beckman Coulter) and analyzed with FlowJo software (Tree Star). Sorting was perfomed on an FACSAria Fusion (Becton Dickinson). Single cell suspensions were incubated with Zombie Aqua (Biolegend) or Viakrome 808 fixable viability dye (Beckman Coulter) diluted 1:250 in PBS for 15 minutes at 4°C. Samples were centrifuges, resuspended in FACS buffer with anti-CD16/32 antibody (Biolegend) diluted 1:50 in FACS buffer, followed by staining with the antibodies for 15 minutes at 4°C.
Instrument	CytoFLEX LX (Beckman Coulter) for FACS, FACSAria Fusion (Becton Dickinson) for cell sorting.
Software	FlowJo version 10.6.2 was used for data analysis.
Cell population abundance	Cell populations were abundant enough for any of the analysis. Approximately over 200 cells of each target cell population were detected from the synovium per mouse and we compiled multiple mice when needed.
Gating strategy	The initial gate on FSC/SSC plots was set to remove cell debris and single cells were gated according to FSC-W and FSC-H. After gating on live cells using ViaKrome 808, target cell populations were gated as depicted in Supplementary Fig. 3c.

☒ Tick this box to confirm that a figure exemplifying the gating strategy is provided in the Supplementary Information.
